# Kinetics of laser-induced melting of thin gold film: How slow can it get?

**DOI:** 10.1126/sciadv.abo2621

**Published:** 2022-09-21

**Authors:** Mikhail I. Arefev, Maxim V. Shugaev, Leonid V. Zhigilei

**Affiliations:** Department of Materials Science and Engineering, University of Virginia, Charlottesville, VA 22904-4745, USA.

## Abstract

Melting is a common and well-studied phenomenon that still reveals new facets when triggered by laser excitation and probed with ultrafast electron diffraction. Recent experimental evidence of anomalously slow nanosecond-scale melting of thin gold films irradiated by femtosecond laser pulses motivates computational efforts aimed at revealing the underlying mechanisms of melting. Atomistic simulations reveal that a combined effect of lattice superheating and relaxation of laser-induced stresses ensures the dominance of the homogeneous melting mechanism at all energies down to the melting threshold and keeps the time scale of melting within ~100 picoseconds. The much longer melting times and the prominent contribution of heterogeneous melting inferred from the experiments cannot be reconciled with the atomistic simulations by any reasonable variation of the electron-phonon coupling strength, thus suggesting the need for further coordinated experimental and theoretical efforts aimed at addressing the mechanisms and kinetics of laser-induced melting in the vicinity of melting threshold.

## Teaser

The melting of Au films in atomistic simulations is ultrafast down to the melting threshold, thus calling for more experiments.

## INTRODUCTION

Melting is a common phase transformation that plays a prominent role in many industrial applications and is widely observed in daily life. While the microscopic mechanisms and kinetics of melting have been the subjects of theoretical, computational, and experimental investigations for over more than a century (*1*–*10*), this well-studied phenomenon still reveals new and not fully understood facets under conditions of ultrashort (femto- or picosecond) pulse laser excitation, when the crystal is driven far from its thermodynamic, mechanical, and electron-phonon equilibrium (*11*–*26*). In particular, a series of time-resolved electron diffraction studies performed for thin (10 to 35 nm) gold (Au) films irradiated by ultrashort laser pulses (*15*–*18*, *20*–*22*, *26*) has provided valuable insights into the kinetics of melting and yet presented new challenges for the theoretical interpretation. The Au films are particularly suitable for analysis of the laser-induced melting due to the relatively weak electron-phonon coupling in Au (*27*–*29*), which leads to an increased time scale of lattice heating and a more apparent, as compared to metals with stronger election-phonon coupling (*29*), separation of nonthermal effects defined by the electronic excitation (*17*, *21*, *25*, *30*) from thermally driven atomic dynamics and phase transformations (*12*–*16*, *18*, *19*, *20*–*22*). Moreover, for films with thicknesses smaller than the range of the ballistic energy transport by the excited electrons (~100 nm for Au) (*27*), a uniform electron temperature distribution throughout the film thickness is established before any substantial lattice heating, making the interpretation of the experimental observations more straightforward.

In the discussion provided below, we take advantage of the uniformity of the laser energy deposition throughout the thickness of the film and convert the values of absorbed laser fluences reported in the literature to the deposited energy density ε, which facilitates the comparison of the results obtained for films of different thickness. The deposited energy density is, in turn, related to the threshold energy density for complete melting ε_m_ = 0.212 MJ/kg evaluated by integration of the temperature-dependent heat capacity *c*_p_(*T*) from 300 K to the melting temperature of *T*_m_ = 1337 K, which yields 29.1 kJ/mol = 0.15 MJ/kg, and adding the enthalpy of melting Δ*H*_m_ = 12.72 kJ/mol = 0.065 MJ/kg (*31*). In the discussion of computational predictions obtained in simulations combining molecular dynamics (MD) method with two-temperature model (TTM) (*13*, *14*, *19*–*21*, *23*), we use the values of ε_m_ evaluated using *c*_p_(*T*), *T*_m_, and Δ*H*_m_ calculated with interatomic potentials used in the simulations.

The results of early electron diffraction measurements performed for 20-nm Au films irradiated by 200-fs laser pulses at absorbed fluences of 119 J/m^2^ (ε = 0.31 MJ/kg = 1.5ε_m_) (*15*) and 137 J/m^2^ (ε = 0.35 MJ/kg = 1.7ε_m_) (*16*) reveal a melting process that starts at about 7 ps and takes approximately 3 ps to complete. These results are consistent with a melting time of about 7 ps reported for 35-nm Au film irradiated by a 90-fs laser pulse at ε = 1.8ε_m_ (*21*). The melting time further shortens as the deposited energy density increases by more than an order of magnitude above ε_m_ (*17*), where the interpretation of the results involves a consideration of the transient bond hardening in Au under conditions of strong (several electron volts) electronic excitation predicted in ab initio calculations (*30*). The decrease of the energy density down to the values approaching ε_m_, on the other hand, leads to a gradual increase in the melting time, e.g., up to ~15 ps for a 10-nm Au film irradiated by a 90-fs laser pulse at ε = 1.1ε_m_ (*20*, *21*).

The observation of rapid melting on the picosecond time scale is consistent with the physical picture of homogeneous melting proceeding through massive nucleation and growth of liquid regions in a crystal superheated up to the limit of thermodynamic stability of the crystal lattice (*1*–*9*). The evaluation of the limit of superheating for Au based on the classical nucleation theory, provided in the Supplementary Materials and illustrated in fig. S1, suggests that the phase transformation should occur within ~10 ps when the temperature reaches the level of T^*^ = 1.25 *T*_m_. The nucleation rate of ~10^35^ s^−1^ m^−3^ and the critical radius of the liquid nucleus of ~0.9 nm (only a few interatomic distances) estimated for T^*^ (*32*), however, suggest that the physical picture of nucleation and growth of spherical liquid regions, assumed in the calculations based on the classical nucleation theory, is unlikely to remain valid at temperatures approaching T^*^. TTM-MD simulations of laser-induced melting of Au films predict a rapid collapse of the crystal lattice proceeding through the simultaneous formation of a large number of interconnected irregularly shaped liquid regions (*13*, *14*, *19*–*21*). Moreover, the simulations reveal that the dynamic relaxation of stresses generated by the fast laser heating can reduce the level of superheating required for the initiation of homogeneous melting down to as low as T^*^ = 1.05 *T*_m_ (*13*).

Another mechanism of melting, alternative to the massive nucleation of liquid regions inside the superheated crystal, is the heterogeneous nucleation of liquid at free surfaces of the irradiated film followed by the propagation of the melting fronts toward the center of the film. While it is reasonable to expect that the contribution of the heterogeneous melting mechanism may increase as the laser fluence decreases and approaches the threshold for complete melting, a quantitative analysis of the kinetics of melting (*32*) and the results of TTM-MD simulations (*14*, *19*–*21*) suggest that, in the case of ultrashort pulse laser interaction with thin Au films, the melting front propagation becomes dominant only below the threshold for complete melting. The superheating that can be created at the threshold for complete melting of a uniformly heated Au film can be estimated with Δ*H*_m_ = 12.72 kJ/mol and *c*_p_(*T*_m_) = 32.3 J/mol K as *T* = *T*_m_ + Δ*H*_m_ /*c*_p_(*T*_m_) = 1.29 *T*_m_, which is higher than the limit of superheating T^*^ for the onset of massive homogeneous nucleation of liquid regions inside the superheated crystal. Moreover, the distance the melting fronts can propagate during the short time the electron-phonon coupling heats the film from *T*_m_ to T^*^ is far below the thickness of the films used in the experiments. The results of TTM-MD simulations performed for 20-nm Au films suggest that the homogeneous nucleation of liquid regions and heterogeneous propagation of melting fronts from the free surfaces make a comparable contribution just above the threshold for complete melting, at the deposited energy density of 1.02ε_m_ (*14*). The melting proceeding through the propagation of melting fronts alone, without the contribution of the homogeneous nucleation, is only observed in simulations of partial (incomplete) melting, at 0.84ε_m_ for 20-nm Au films (*14*, *19*) and at 0.97ε_m_ for a 10-nm film (*21*).

The results of recent ultrafast electron diffraction experiments performed for single-crystal 35-nm Au films irradiated by 130-fs laser pulses (*22*), however, are in sharp contrast with the computational predictions and put the logic of the discussion provided above into question. In particular, the experimental data suggest a large increase in the melting time as the deposited energy density decreases below 0.4 MJ/kg = 1.9ε_m_, which is interpreted as an indication of the transition to the regime of heterogeneous melting. At an energy density of 0.36 MJ/kg = 1.7ε_m_, the presence of the diffraction peaks corresponding to the crystalline Au is reported to persist up to 800 ps, and the melting time in excess of 2 ns is reported for 0.317 MJ/kg = 1.5ε_m_. The apparent disagreement with the results of TTM-MD simulations, where the melting time remains below 100 ps as the deposited energy density decreases down to ε_m_ (*14*, *19*–*21*, *23*, *33*), has been attributed to the inaccuracies of interatomic potentials and overestimation of the strength of the electron-phonon coupling (*22*, *26*). The observation of slower, as compared to earlier studies (*15*–*18*, *20*, *21*), melting process has been used (*26*, *34*) as experimental evidence in favor of the weakest electron temperature dependence of the electron-phonon coupling in Au (*34*) out of various dependences suggested in theoretical studies (*28*, *29*, *34*–*40*).

Here, we report results of a new series of TTM-MD simulations designed to reproduce the experimental conditions of (*22*) and to check the hypothesis that the discrepancy between the time of complete melting observed in the experiments and predicted in earlier simulations can be eliminated by using an improved interatomic potential and assuming a lower strength of the electron-phonon coupling (*22*, *26*). The melting process is tracked by performing structural analysis of atomic configurations and calculating two-dimensional (2D) diffraction patterns for different moments of time during the simulations. We find that the long melting times in the vicinity of the melting threshold and the contribution of the heterogeneous melting inferred from the experiments cannot be reconciled with the atomistic simulations by any reasonable variation of the electron-phonon coupling parameter, thus suggesting the need for further coordinated experimental and theoretical efforts aimed at addressing the mechanisms and kinetics of laser-induced melting.

## RESULTS

The TTM-MD simulations are performed for single-crystal 35-nm Au films irradiated by 130-fs laser pulses at the absorbed energy density ranging from 0.18 to 1.17 MJ/kg. These conditions reproduce those used in the experimental probing of the melting process reported in (*22*). The focus of this study is on the exploration of melting mechanisms at energy densities approaching the threshold for complete melting, where an anomalously slow nanosecond-scale melting is reported (*22*, *26*). Therefore, we only briefly discuss the high energy density regime, where a rapid homogeneous melting is observed in both simulations and experiments, and then provide a more detailed analysis of the mechanisms and kinetics of melting in the vicinity of the threshold for complete melting of the film.

### Rapid homogeneous melting at 1.17 MJ/kg (5.5 ε_m_)

The highest absorbed energy density considered in the simulations is 1.17 MJ/kg, which is more than five times higher than the threshold for complete melting of the film. At this energy density, the TTM-MD simulation performed with the electron temperature-dependent electron-phonon coupling parameter *g*(*T*_e_) (*28*, *29*) predicts that the lattice temperature *T*_l_ (*41*) exceeds zero-pressure melting temperature *T*_m_ at ~1 ps and reaches 2 *T*_m_ at ~2.3 ps and 3 *T*_m_ at ~4.9 ps (fig. S2). The rapid heating leads to the massive homogeneous nucleation of liquid regions inside the superheated crystal and the complete melting by 5 ps after the laser pulse. The time for the complete melting predicted in the simulation is substantially shorter than ~15 ps reported in (*22*), where a constant (temperature-independent) value of the electron-phonon coupling parameter of *g* = 4.9 × 10^16^ Wm^−3^ K^−1^ is suggested to provide a good fit of the experimental observations. The TTM-MD simulation performed with this value of *g* does predict a longer ~11.4 ps time for the complete melting due to the slower electron-phonon equilibration. Since this time is still below the experimental value, we performed additional TTM-MD simulations and determined that the experimental time for the complete melting can be reproduced with an electron-phonon coupling parameter of 2.8 × 10^16^ Wm^−3^ K^−1^.

### Melting at 0.36 MJ/kg (1.7 ε_m_) and 0.317 MJ/kg (1.5 ε_m_)

The complete melting time measured for single-crystal 35-nm Au films in experiments reported in (*22*) exhibits a gradual increase from 15 to 45 ps as the deposited energy density ε decreases from 1.17 to 0.4 MJ/kg, followed by a marked rise to 800 ps at ε = 0.36 MJ/kg and to 2500 ps at ε = 0.317 MJ/kg. The abrupt increase of the melting time is attributed to the transition from homogeneous to heterogeneous melting regimes occurring at ε ≈ 0.4 MJ/kg ≈ 1.9 ε_m_. To verify this hypothesis, we perform a detailed computational analysis of the melting process at the same energy densities as those applied in the experiments.

**Fig. 1.**
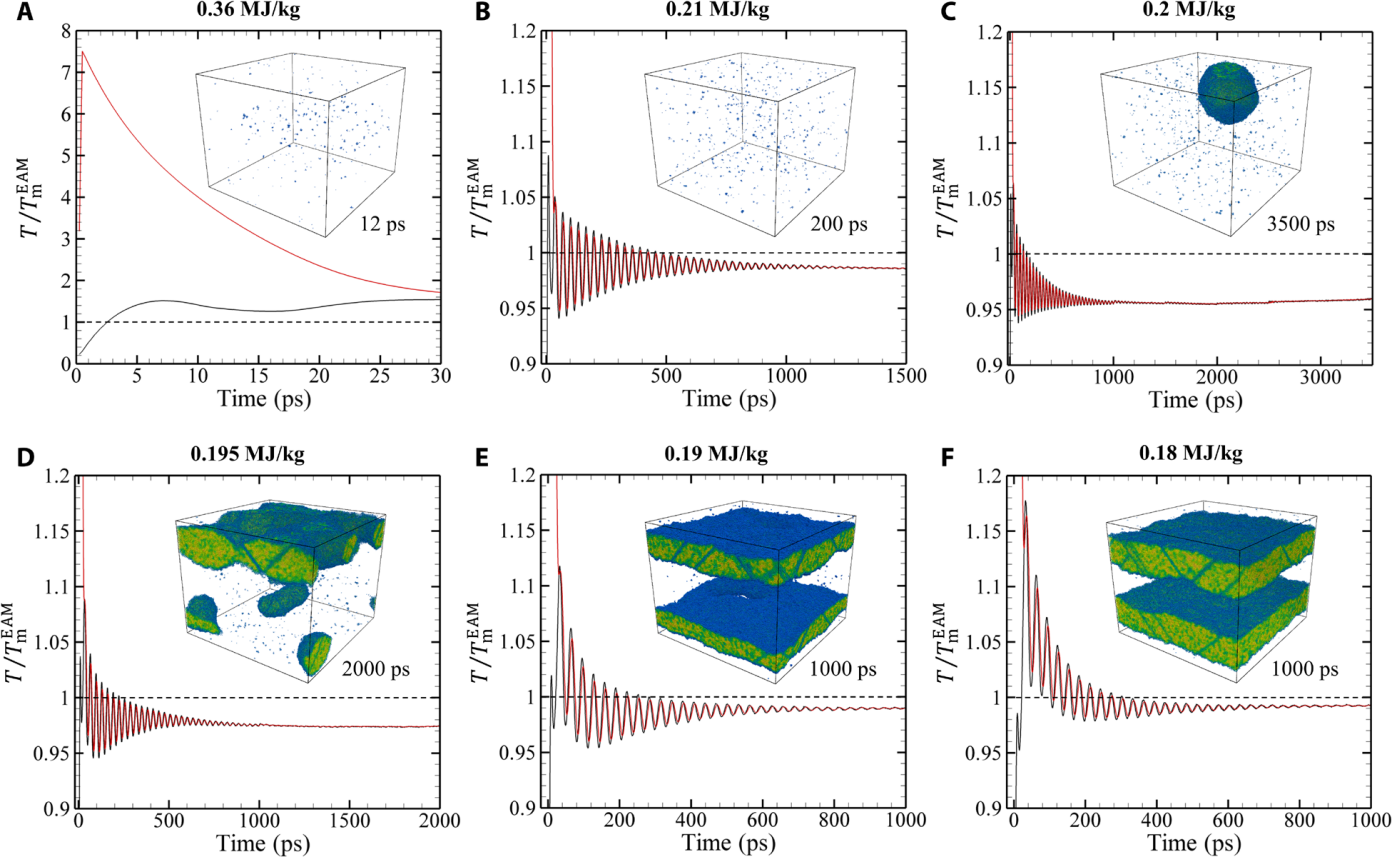
Lattice and electron temperature profiles. The evolution of the average lattice (black) and electron (red) temperatures in 35-nm Au films irradiated by 130-fs laser pulses at absorbed energy densities of 0.36 (**A**), 0.21 (**B**), 0.20 (**C**), 0.195 (**D**), 0.19 (**E**), and 0.18 MJ/kg (**F**). The temperatures are normalized by the equilibrium melting temperature at zero pressure of the model material, $T_{m}^{\text{EAM}}=1331\;K$. Insets show snapshots of atomic configuration at times indicated next to the insets.

In the TTM-MD simulation performed at ε = 0.36 MJ/kg, the average lattice temperature of the film exceeds *T*_m_ by ~2.5 ps, reaches 1.5 *T*_m_ at 7 ps, exhibits a transient decrease down to 1.26 *T*_m_ at 16 ps, and then undergoes periodic oscillations (Fig. 1A). The transient decrease of the lattice temperature takes place during the time when *T*_e_ > *T*_l_, and the energy is still transferred from the electrons to the lattice. This decrease is defined by the combined contribution of two factors.

The first factor is the melting process that occurs on the same time scale as the transient decrease of *T*_l_ and leads to the conversion of a part of the thermal energy to the latent heat of melting. The temperature drop associated with melting alone can be quantified as Δ*T* ≈ Δ*H*_m_/*c*_p_(*T*_m_) = 0.29*T*_m_ for the experimental properties of Au and $\Delta T\approx \Delta H_{m}^{\text{EAM}}/c_{p}^{\text{TTM}-\text{MD}}(T_{m}^{\text{EAM}})=0.31T_{m}^{\text{EAM}}$ for the model system described in Materials and Methods.

**Fig. 2.**
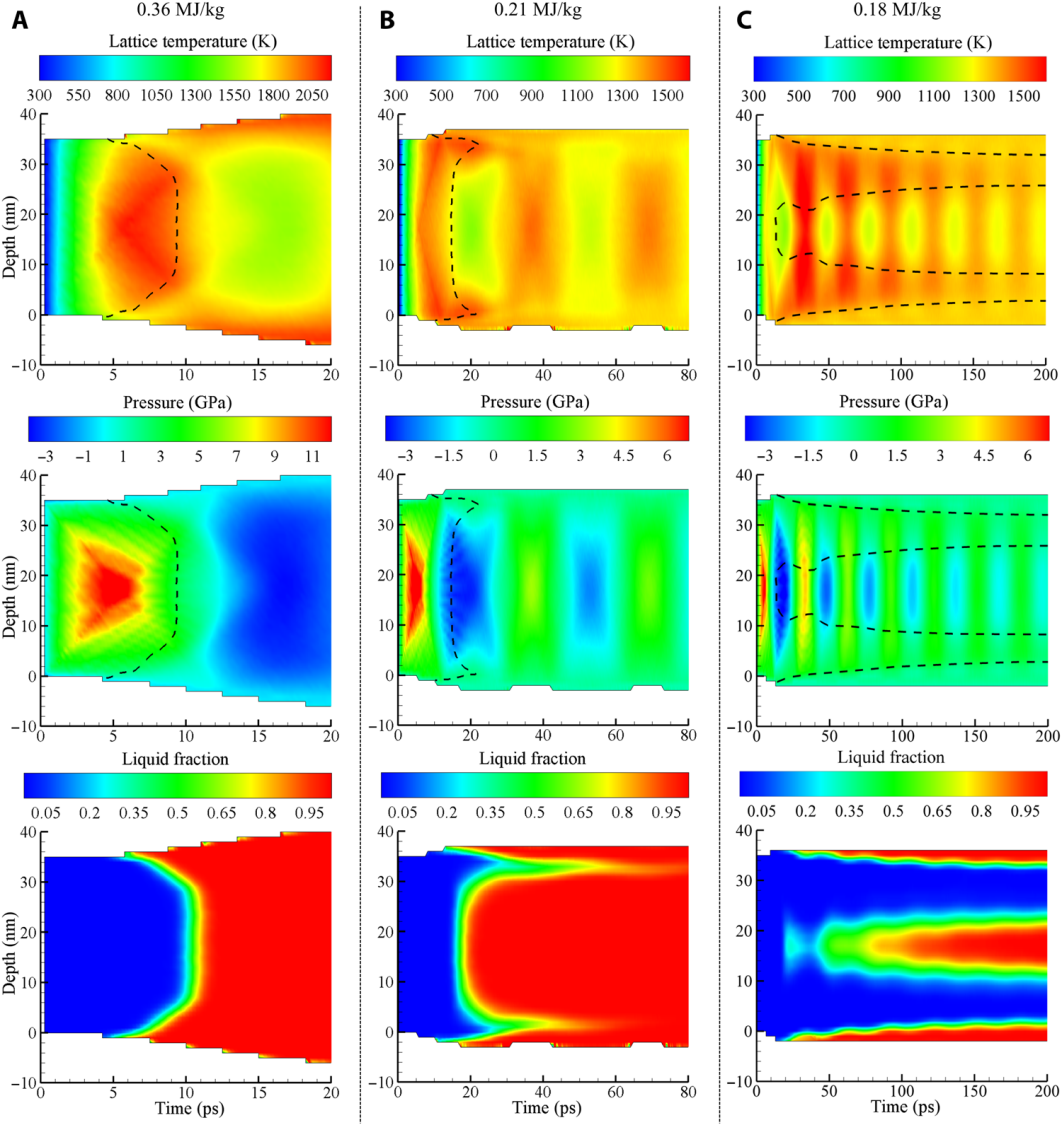
Temporal and spatial evolution of temperature, pressure, and phase state in the irradiated films. Contour plots of the lattice temperature (top), pressure (middle), and the fraction of atoms in the liquid phase (bottom) in Au films irradiated by 130-fs laser pulses at absorbed energy densities of 0.36 (**A**), 0.21 (**B**), and 0.18 MJ/kg (**C**). The dashed lines in the temperature and pressure plots show the location and time of the start of the melting process defined as the place where the liquid fraction is equal to 0.05.

The second factor is related to the relaxation of large compressive stresses generated in the central part of the film during the initial stage of the electron-phonon equilibration, on a time scale shorter than that of the film expansion. The buildup and relaxation of the compressive stresses can be seen in the pressure contour plot (*42*) shown in Fig. 2A. The pressure reaches its maximum level of more than 12 GPa by about 5 ps and is erased by two unloading waves propagating from the free surfaces of the film. The unloading waves turn the compression into tension, and the rapid expansion of the film leads to a substantial temperature drop. The connection between the pressure and temperature variations in a material undergoing rapid adiabatic/isentropic expansion or compression can be expressed as (∂*T*/∂*P*)_S_ = *VT*α_V_/*c*_p_ (*13*, *43*), where *V* is the molar volume and α_V_ is the volumetric coefficient of thermal expansion. It has been demonstrated (*13*) that the integration of this simple equation provides a reasonable semiquantitative description of the connection between the pressure and temperature variations in metal films irradiated by short laser pulses.

The generation and relaxation of laser-induced pressure are also playing an important role in defining the kinetics of the melting process. As can be seen from the contour plot of the phase state of Au in Fig. 2A, the melting starts at the two surfaces as soon as the temperature exceeds *T*_m_. The crystal structure in the central part of the film, however, survives the brief superheating up to the maximum level of 1.62 *T*_m_ at a time when the pressure reaches its maximum level of about 12 GPa. This observation does not contradict the estimation of the maximum level of superheating discussed in Introduction, T^*^ ≤ 1.25 *T*_m_, since the melting temperature increases with increasing pressure according to the Clapeyron equation, (*∂T*/*∂P*)_m_ ≈ Δ*V*_m_*T*_m_/Δ*H*_m_. The slope of the melting curve in the vicinity of zero pressure is 54 K/GPa when evaluated with experimental parameters of Au and is 68 K/GPa for the model system. In both cases, (*∂T*/*∂P*)_m_ is substantially larger than (*∂T*/*∂P*)_S_ evaluated for the crystalline Au at *T*_m_ (by a factor of 2 for real Au and a factor of 1.7 for the model Au). As a result, the central part of the film does not melt when the maximum temperature is reached there at *P* ≈ 12 GPa but undergoes a rapid homogeneous melting upon the unloading, when the cooling due to the adiabatic expansion cannot compensate for the rapid drop of the melting temperature. The homogeneous nucleation of liquid regions is also assisted by the anisotropic lattice distortions associated with the uniaxial expansion of the film in the direction normal to the free surfaces, which further reduce the stability of the crystal lattice against melting (*13*).

**Fig. 3.**
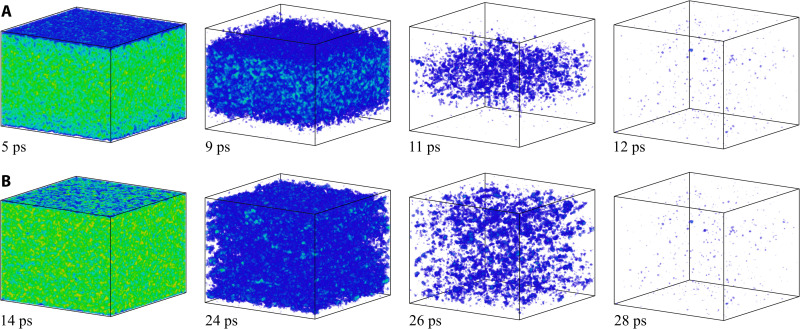
Atomic snapshots illustrating the melting process of a Au film irradiated at an absorbed energy density of 0.36 MJ/kg. The snapshots are from TTM-MD simulations performed with *g*(*T*_e_) from (*29*) (**A**) and *g* = 2.7 × 10^16^ Wm^−3^ K^−1^ suggested for ε = 0.36 MJ/kg in (*22*) (**B**). The atoms that belong to the liquid phase are blanked, and the remaining atoms are colored according to the local order parameter, with color variation from blue to red for the local order parameter ranging from 0.04 to 1.

The film is fully melted by 12 ps, as can be seen from the contour plots in Fig. 2A and the snapshots of atomic configurations in Fig. 3A, where only the atoms with local crystalline surroundings are shown. The time for complete melting predicted in the simulation is much shorter than 800 ps observed in the ultrafast electron diffraction experiments at the same absorbed energy density (*22*). When the TTM-MD simulation is repeated with a constant electron-phonon coupling parameter suggested in (*22*), *g* = 2.7 × 10^16^ Wm^−3^ K^−1^, the energy transfer to the lattice is slower, and the onset of melting is delayed to about 20 ps, as can be seen from the snapshots shown in Fig. 3B. The melting process, however, remains qualitatively similar to that discussed above for a simulation performed with *g*(*T*_e_) (*28*, *29*). The melting proceeds mainly through the homogeneous nucleation and growth of liquid regions inside the superheated crystal, leading to the complete melting within about 7 ps after the start of the melting process (Fig. 3B). The complete melting within 27 ps after the irradiation is still very far from the experimental time of 800 ps. To reproduce the experimental time for the complete melting in the TTM-MD simulations, the value of *g* should be reduced down to 0.074 × 10^16^ Wm^−3^ K^−1^, which is more than an order of magnitude lower than the lowest values evaluated from the results of optical pump-probe measurements performed for Au targets (*27*, *44*–*51*).

The TTM-MD simulations were also performed for a lower deposited energy density of 0.317 MJ/kg (1.5 ε_m_), for which an anomalously slow melting time of 2.5 ns was reported and attributed to the heterogeneous melting mechanism in (*22*). The melting time with a constant value of *g* = 2.6 × 10^16^ Wm^−3^ K^−1^, based on the linear dependence of *g* on ε suggested in (*22*), is found to be only 32 ps at this energy density, and the melting process is still dominated by the homogeneous nucleation of liquid regions inside the superheated film, similar to that discussed above for the higher energy density (0.36 MJ/kg). The value of *g* that has to be assumed to reproduce the experimental melting time of 2.5 ns is found to be 0.027 × 10^16^ Wm^−3^ K^−1^, i.e., two orders of magnitude lower than the typical values measured in the optical pump-probe experiments.

### Melting in a close vicinity of ε_m_: How slow can it get?

Given the large mismatch between the times for complete melting predicted in the TTM-MD simulations and measured in the ultrafast electron diffraction probing of the melting process at nominally the same energy densities deposited by the laser pulses, the natural question is whether it is possible to reproduce the nanosecond-scale complete melting in the simulations at any level of laser excitation. We find that the time scale of melting is gradually increasing with decreasing laser energy density, and the slowest complete melting is observed at the lowest energy where the complete melting is still observed, 0.21 MJ/kg (0.99 ε_m_).

**Fig. 4.**
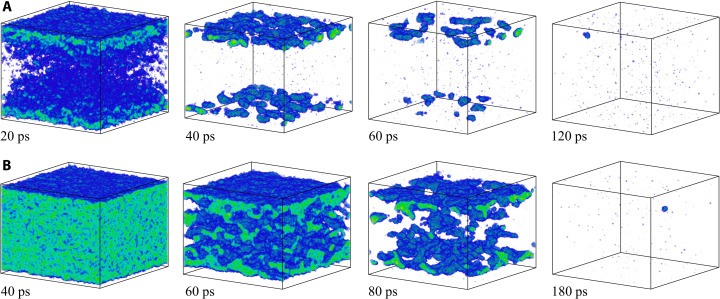
Atomic snapshots illustrating the melting process of a Au film irradiated at an absorbed energy density of 0.21 MJ/kg. The snapshots are from TTM-MD simulations performed with *g*(*T*_e_) from (*29*) (**A**) and *g* = 2.3 × 10^16^ Wm^−3^ K^−1^ suggested for ε = 0.21 MJ/kg in (*22*) (**B**). The coloring scheme is the same as in Fig. 3, and the atoms that belong to the liquid phase are blanked.

The snapshots from the simulation performed just 1% below the threshold for complete melting are shown in Fig. 4A. The melting starts from the two surfaces of the film, but the propagation of the melting fronts makes rather limited contribution to the overall melting process, which is still dominated by the homogeneous nucleation and growth of liquid regions inside the film. Similar to the melting process at 0.36 MJ/kg discussed above, the nucleation of liquid regions takes place upon the unloading of the initial compressive stresses generated by the rapid energy deposition, and the conditions for the homogeneous nucleation are defined by the interplay of the transient cooling and the depression of the melting temperature by the anisotropic tensile stresses produced by the uniaxial expansion of the film. The central part of the film is largely melted by 20 ps, as can be seen from the contour plot of the liquid fraction in Fig. 2B. The remaining crystalline islands, sandwiched between the heterogeneously melted surface regions and the homogeneously melted central part of the film, are slowly shrinking during the following 100 ps and completely disappear by 130 ps. The average temperature of the fully molten film (Fig. 1B) exhibits gradually decaying oscillations reflecting the elastic vibration of the film and saturates at a level of about 0.99 *T*_m_, which is expected since $\Delta T=(\varepsilon_{m}-\varepsilon )/c_{p}^{\text{TTM}-\text{MD},l}(T_{m}^{\text{EAM}})\approx 0.01T_{m}^{\text{EAM}}$ for ε = 0.21 MJ/kg.

The simulation illustrated in Figs. 1B, 2B, and 4A and discussed above is performed with *g*(*T*_e_) (*28*, *29*). When the simulation is repeated with a constant value of *g* = 2.3 × 10^16^ Wm^−3^ K^−1^ taken from the linear *g*(ε) dependence suggested in (*22*), the melting is delayed but proceeds through the similar mechanisms. The substantially weaker effective strength of the electron-phonon coupling leads to a slower heating of the lattice and a reduced magnitude of laser-induced stresses. In particular, the maximum tensile stresses in the central part of the film are reduced from about −3.6 to about −1.7 GPa (*42*), which is not sufficient for triggering the homogeneous melting during the first cycle of pressure oscillations. By about 40 ps, the temperature reaches the level of about 1.2 *T*_m_, and the homogeneous nucleation of liquid regions takes place when the tensile stresses are produced for the second time in the central part of the film (fig. S3). The whole film is fully melted by 190 ps after the laser pulse, as can be seen in Fig. 4B.

**Fig. 5.**
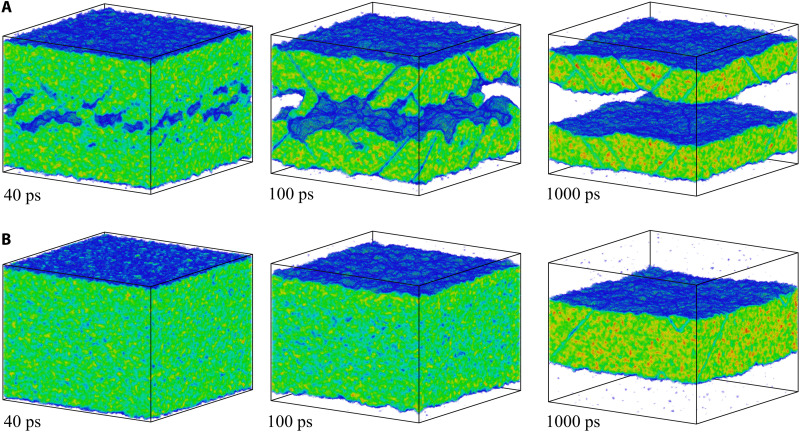
Atomic snapshots illustrating the melting process of a Au film irradiated at an absorbed energy density of 0.18 MJ/kg. The snapshots are from TTM-MD simulations performed with *g*(*T*_e_) from (*29*) (**A**) and *g* = 2.2 × 10^16^ Wm^−3^ K^−1^ suggested for ε = 0.18 MJ/kg in (*22*) (**B**). The coloring scheme is the same as in Fig. 3, and the atoms that belong to the liquid phase are blanked.

Further reduction of the deposited laser energy below 0.21 MJ/kg leads to the transition to the regime of incomplete melting, when the molten material and crystalline regions coexist in the final state of the system. The results of the simulations performed at deposited energies of 0.18, 0.19, 0.195, and 0.2 MJ/kg are illustrated by spatially averaged temperature profiles and snapshots of final configurations in Fig. 1 (C to F). In all of these simulations, the relaxation of laser-induced stresses leads to the homogeneous nucleation of liquid regions in the central parts of the films and the formation of solid regions sandwiched between the homogeneously and heterogeneously melted parts of the films. In particular, at ε = 0.18 MJ/kg (0.85 ε*_m_*) illustrated by Figs. 1F, 2C, and 5A, the melting starts with propagation of two melting fronts from the free surfaces of the film, and the new liquid regions are generated at about 20 ps, when the tensile stresses of −3.9 GPa (*42*) are produced in the middle of the film by the unloading waves. This level of tensile stresses can reduce the melting temperature of the model material by 267 K (0.2 *T*_m_) according to the Clapeyron equation, (*∂T*/*∂P*)_m_ ≈ 68 K/GPa, and produce an additional reduction of the crystal stability against melting through the anisotropic (uniaxial) lattice expansion (*13*). The new liquid regions grow, coalesce, and form a continuous liquid layer at the middle of the film by about 100 ps (Fig. 5A). The following propagation of the four solid-liquid interfaces leads to the eventual melting of approximately 46% of the film material before the temperature in the system stabilizes at 0.99 *T*_m_ (Fig. 1F), and the melting stops. The observation that the final temperature is slightly lower than *T*_m_ can be explained by the residual stresses that cannot fully relax through the uniaxial expansion and plastic deformation of the crystalline layers. As a result, the equilibrium between the molten material and the strained crystalline films shifts to a lower temperature with respect to that for a fully relaxed crystal.

The melting process at higher energies approaching the threshold for complete melting is similar to that described above, except that the crystalline layers become thinner (e.g., Fig. 1E for 0.19 MJ/kg) and disintegrate into separate crystalline regions. At 0.195 MJ/kg, only one of the two crystalline layers disintegrates into separate regions, while at 0.2 MJ/kg, both layers disintegrate. The disintegration of continuous crystalline layers leads to a substantial increase in the fraction of liquid and a decrease in the average temperature of the film (Fig. 1, C and D). These observations can be explained by considering the condition for equilibrium between a spherical crystalline region and the surrounding undercooled molten material (*19*). For a given undercooling Δ*T* below *T*_m_, i.e., at *T* = *T*_m_ − Δ*T*, the solid regions with diameters below D^*^ = (4γ_LC_*T*_m_)/(∆*H_m_*∆*T*) are thermodynamically unstable and should melt out, even though *T* < *T*_m_.

Taking the case of 0.20 MJ/kg as an example, both crystalline layers formed at the early stage of the melting process disintegrate during the first several hundreds of picoseconds after the laser pulse (fig. S4). Most of the fragments with diameters much smaller than D^*^ disappear within the first nanosecond after the laser pulse. By the time of 1 ns, only three largest crystalline regions with effective diameters (*52*) of 9.8, 10.1, and 18.4 nm are present in the molten film. Two of these crystalline regions are smaller than D^*^=11.2 nm calculated for the temperature established in the film by 1 ns, *T* = 0.96 *T*_m_ (Fig. 1C). These smaller regions shrink and completely disappear by 2 ns, while the largest region slowly grows in size. The disappearance of the smaller crystalline regions leads to a small additional decrease in temperature (Fig. 1C) and increase in the total fraction of liquid in the film (fig. S5). After 2 ns, these trends are reversed, as the largest and thermodynamically stable crystalline region grows in size.

Similar to the simulations performed at higher energy densities, the application of a constant value of the electron-phonon coupling parameter in lieu of *g*(*T*_e_) in the simulations performed for ε in the vicinity of ε_m_ leads to some changes in the details of the melting process but does not substantially affect its overall kinetics. For example, in a simulation performed for ε = 0.18 MJ/kg with a constant value of *g* = 2.2 × 10^16^ W m^−3^ K^−1^ obtained from the linear *g*(ε) dependence (*22*), the heating rate and the magnitude of pressure oscillations are lower, and the homogeneous nucleation of liquid regions in the central part of the film does not occur (Fig. 5B). As a result, the melting proceeds through the propagation of two melting fronts from the surfaces of the film, forming a single crystalline layer in the middle of the partially molten film. The total fraction of the crystalline material and the melting time, however, do not significantly differ in the two simulations illustrated in Fig. 5 (A and B).

Overall, we can conclude that the slowest complete melting of a 35-nm-thick Au film irradiated by a femtosecond laser pulse is observed just below the energy density threshold for complete melting (at ε = 0.21 MJ/kg = 0.99 ε_m_). It takes less than 130 ps when *g*(*T*_e_) (*28*, *29*) is assumed in the simulation and less than 190 ps when a constant value from *g*(ε) (*22*) is used. At lower ε, the melting is incomplete, while at higher ε, the melting time is shorter.

### Diffraction patterns

**Fig. 6.**
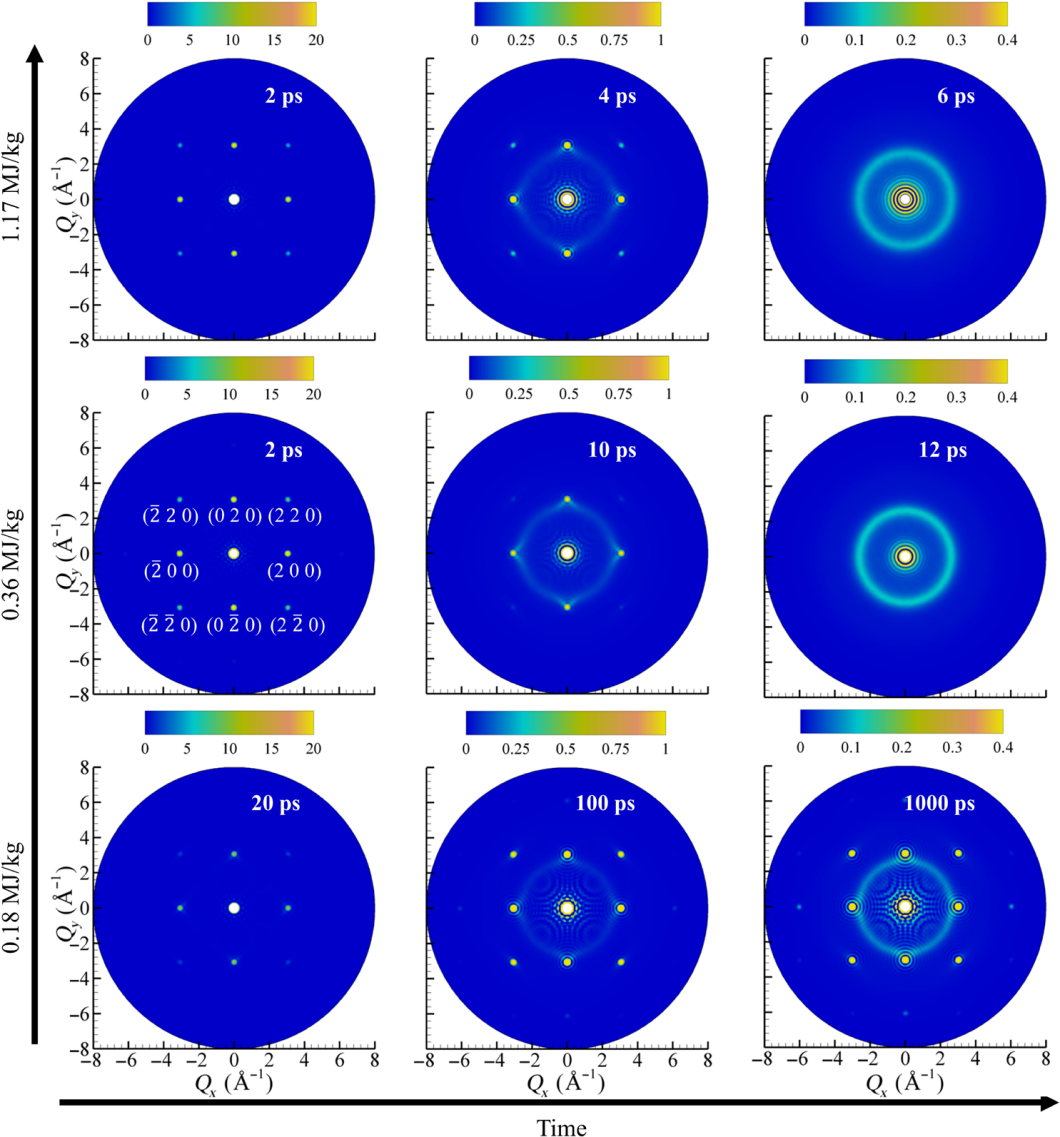
Diffraction patterns predicted in the simulations. 2D electron diffraction patterns (scattering intensity) calculated for several atomic configurations generated in the simulations for melting of Au films irradiated by 130-fs laser pulses at absorbed energy densities of 1.17 MJ/kg (top row), 0.36 MJ/kg (middle row), and 0.18 MJ/kg (bottom row). The spurious ripples in the vicinity of the central peak are due to the finite size of the system magnified by scattering factors for electron diffraction used in the calculations.

To provide a direct connection between the computational predictions and the results of experimental electron diffraction probing of the melting process, series of 2D diffraction patterns (Fig. 6) are calculated for atomic configurations generated in the TTM-MD simulations performed at the same energy densities of 0.18, 0.36, and 1.17 MJ/kg as those investigated in (*22*).

At the highest energy density of 1.17 MJ/kg, the three diffraction patterns show clear crystalline peaks at 2 ps, the appearance of a weak liquid diffraction ring coexisting with distinct crystalline peaks at 4 ps, and the complete disappearance of the crystalline peaks by 6 ps. The evolution of the diffraction pattern is similar to that observed at the same energy density in the experiments, although the time scale is different. The onset of melting, signified by the appearance of a weak diffraction ring, is observed at about 7 ps, and the crystalline peaks disappear only by about 17 ps (*22*).

At ε = 0.36 MJ/kg, a pronounced liquid ring can be seen in the experimental diffraction pattern as early as 20 ps, suggesting that the temperature of the film has already surpassed the melting temperature by this time. Unexpectedly, however, the crystalline peaks are still clearly visible in the experimental diffraction patterns for the following several hundreds of picoseconds, and the melting process is reported to be completed only by 800 ps (*22*). In contrast, the simulated diffraction patterns (Fig. 6) reveal the complete disappearance of the crystalline Bragg peaks by 12 ps, i.e., within several picoseconds after the start of the homogeneous melting process.

At ε = 0.18 MJ/kg, i.e., at the energy density 15% below the threshold for the complete melting of the Au film, both the simulated and experimental diffraction patterns exhibit the appearance of a liquid ring by 100 ps and the formation of a long-lasting state of crystal-liquid coexistence by 1 ns. At a longer time scale, beyond 1 ns, the simulated diffraction pattern does not change, while the experimental patterns exhibit a substantial weakening of both the liquid ring and the crystalline peaks between 1 and 3 ns, suggesting the presence of slow processes not observed in the simulations. Given the small thickness of the remaining crystalline layers, e.g., Fig. 5A, one can speculate that the slow dynamics observed for the partially melted system may involve the disintegration of the crystalline layers into separate regions, followed by slow shape evolution and rotation of these crystalline regions.

**Fig. 7.**
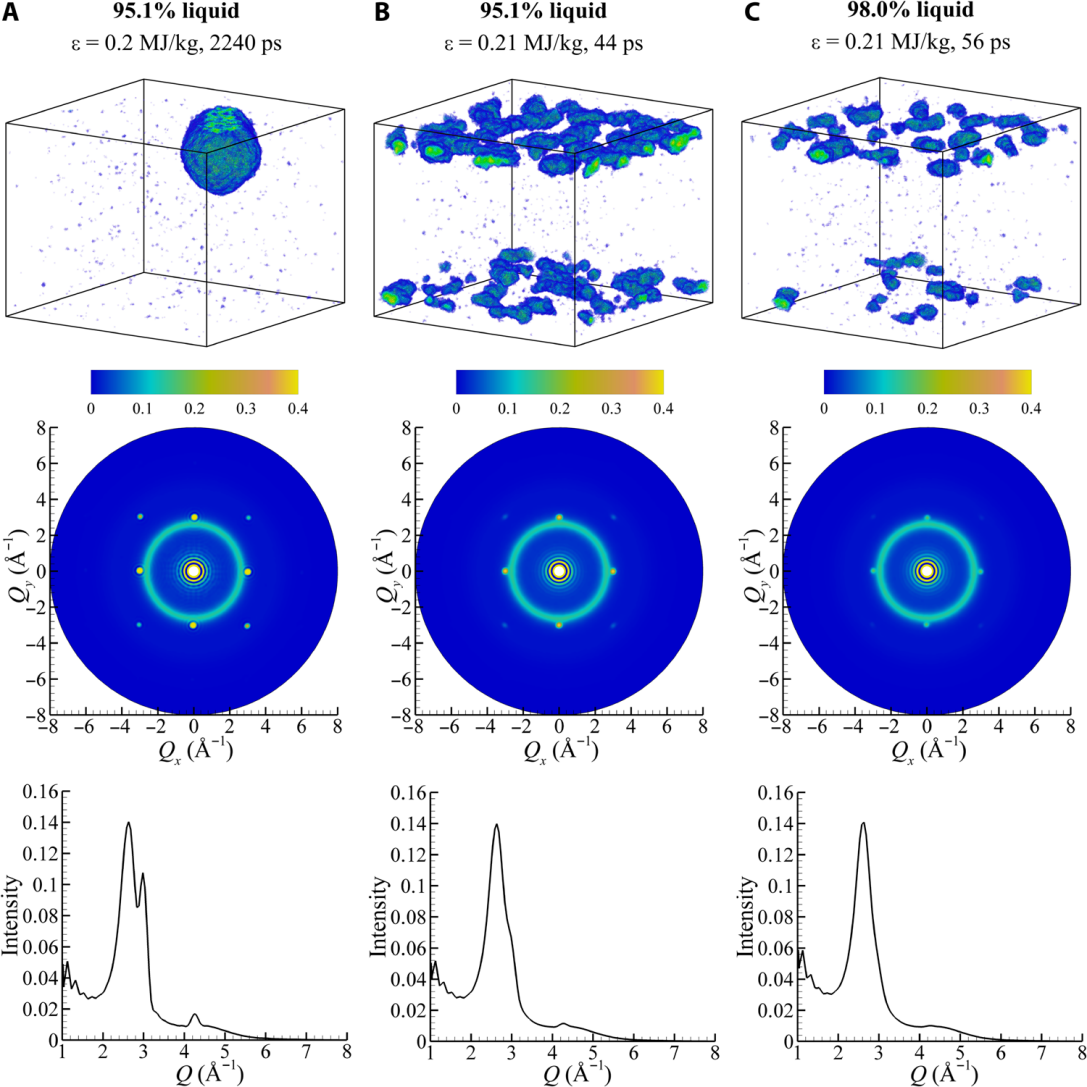
Evaluation of the minimum amount of the crystal phase in the films undergoing rapid laser-induced melting that can be detected in ultrafast electron diffraction experiments. The snapshots of atomic configurations (top row), 2D diffraction patterns (middle row), and radially averaged diffraction profiles (bottom row) are obtained in the simulations of laser-induced melting of Au films irradiated by femtosecond laser pulses at absorbed energy densities of ε = 0.2 MJ/kg (**A**) and ε = 0.21 MJ/kg (**B** and **C**). Only the atoms that belong to the remaining crystalline regions are shown in the snapshots and are colored as described in captions of Figs. 3 to 5. The times chosen for the analysis are indicated at the top of the figure. These times are selected so that the fractions of atoms in the crystalline regions are 4.9% in (A) and (B) and 2% in (C).

The calculation of the diffraction patterns makes it possible to establish the minimum fraction of atoms in the crystalline regions of the film undergoing the laser-induced melting that can still be detected in ultrafast electron diffraction experiments. Before defining the threshold fraction of the crystal phase that matches the definition of the complete melting in experiments, we note that there is no direct one-to-one relationship between the fraction of the crystal phase in the film and the intensity of the crystalline Bragg peaks in the diffraction pattern. The intensity of the crystalline peaks is sensitive to the size distribution of the crystalline regions, as illustrated by the diffraction patterns shown in Fig. 7 (A and B) for two atomic configurations with the same total fraction of the crystal-phase atoms, 4.9%, but with very different size distributions of the crystalline regions. The crystalline peaks in the diffraction pattern calculated for a film where the crystal phase is present in a single region with an effective diameter (*52*) of 20.1 nm (Fig. 7A) are much stronger than those present in the diffraction pattern calculated for a configuration with many small crystalline domains (Fig. 7B). This observation has implications for the mechanistic interpretation of the results of diffraction probing of melting. In particular, it suggests that the homogeneous and heterogeneous melting mechanisms are likely to be distinguishable based on detailed analysis of the evolutions of the diffraction patterns.

**Fig. 8.**
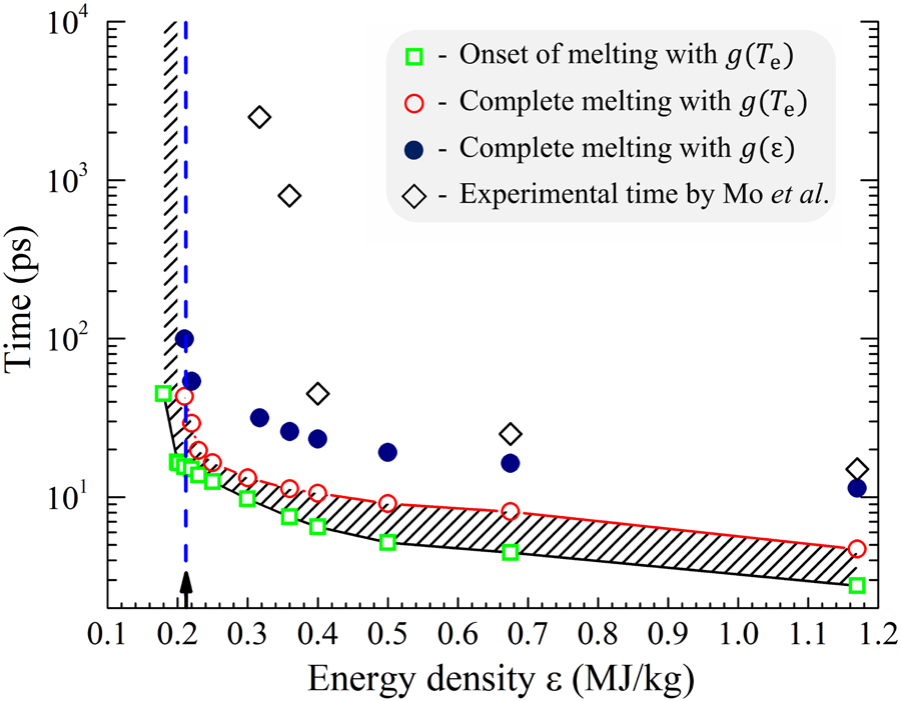
Melting times predicted in TTM-MD simulation and measured in ultrafast electron diffraction experiments. The results of modeling and experiments are shown for 35-nm Au films irradiated by 130-fs laser pulses at different absorbed energy densities ε. Green squares and red circles mark the onset of the melting process (10% of material is molten) and the time of complete melting (98% of material is liquid) predicted in TTM-MD simulations performed with electron temperature-dependent electron-phonon coupling parameter, *g*(*T*_e_) (*28*, *29*), with the duration of melting shown by the shaded region. The blue solid circles mark the time of complete melting predicted in TTM-MD simulations performed with constant (temperature-independent) values of *g* obtained from the linear *g*(ε) dependence suggested in (*22*). The black diamonds mark the time of complete melting measured in the ultrafast electron diffraction experiments reported in (*22*). The vertical blue dashed line shows the theoretical threshold for complete melting, ε_m_.

As the fraction of atoms in the crystalline regions decreases down to 2%, the intensity of the crystalline peaks decreases down to a level comparable to that of the liquid ring, as exemplified by the diffraction pattern shown in Fig. 7C. The maximum intensity of the crystalline peaks from the {200} family of planes in this pattern is only 21% higher than the maximum intensity of the liquid ring, and it drops to a level 40% below the intensity of the liquid ring when the fraction of atoms in the crystalline regions decreases down to 1% in this simulation. While the crystalline peaks are still visible in the calculated diffraction pattern shown in Fig. 7C, these peaks would be difficult to identify from 2D diffraction patterns obtained in ultrafast electron diffraction experiments, where the levels of noise are usually comparable to the intensity of the liquid ring. On the basis of these considerations, we define the time of complete melting in the simulations as a time when the fraction of atoms in the crystalline regions remaining in the film drops below 2%. We note, however, that reducing this threshold level down to 1% would not result in any noticeable changes in the melting times plotted in Fig. 8 and listed in table S1.

Overall, the characteristic times of the onset and completion of the melting process elucidated based on the analysis of the diffraction patterns are consistent with those obtained from direct structural analysis of atomic configurations and counting of atoms with liquid-like local environment. The discrepancy between the TTM-MD simulations and experiments, therefore, cannot be attributed to the ambiguity in the interpretation of the diffraction patterns. Other factors that may be responsible for the large mismatch in the computational and experimental time scales of melting should be considered and are briefly discussed below.

## DISCUSSION

The melting times predicted in the TTM-MD simulations are summarized and compared to the results of ultrafast electron diffraction experiments (*22*) in Fig. 8. The melting observed in the simulations occurs within the time range shown by the shaded area outlined by the black and red lines marking the start and the end of the melting process, respectively. The melting process occurs between 3 and 5 ps at ε = 1.17 MJ/kg = 5.5 ε_m_, and the time scale of melting shifts gradually to longer times as the energy deposited by the laser pulse decreases. The slowest complete melting is observed in a simulation performed at ε = 0.21 MJ/kg = 0.99 ε*_m_*, and it occurs between 16 and 56 ps. By 56 ps, the fraction of the solid phase drops below 2%, which is identified above as the detection limit in diffraction experiments. The small crystalline clusters present at 56 ps continuously shrink and completely disappear by ~130 ps (Fig. 4A). The experimental melting times, shown by open diamonds in Fig. 8, are consistently higher than the computational predictions. Moreover, a notable feature of the experimental data that is not reproduced in the simulations is a sharp (more than an order of magnitude) increase of the melting time when the deposited energy density decreases below 0.4 MJ/kg = 1.9 ε_m_. The factors that may be responsible for the apparent mismatch between the experimental observations and the computational predictions are considered below.

### Interatomic potential and bond hardening

The embedded-atom method (EAM) potential for Au (*53*) used in the present study is providing an adequate description of the key thermodynamic properties of real Au relevant to the melting process. As detailed in Materials and Methods, the melting temperature and the latent heat of melting are within 1% from the experimental values. Moreover, the evaluation of the energy density for the complete melting performed with experimental parameters for Au (*31*) and with parameters predicted by the EAM potential yields the values that coincide down to the third significant digit, ε_m_ = 0.212 MJ/kg. Therefore, any discrepancies between the experimental data and computational predictions are unlikely to be related to inaccuracies of the interatomic potential.

The TTM-MD simulations reported in the present paper do not account for the effect of the electronic excitation on the interatomic interactions. It has been predicted in ab initio electronic structure calculations (*21*, *30*) that the excitation of *d* band electrons in Au reduces the screening between the nuclei and leads to the effective “hardening” of the lattice, which manifests itself in an increase in the Debye temperature, elastic constants, and melting temperature. These theoretical predictions have been invoked in interpretation of the results of electron diffraction probing of the structural dynamics in 20-nm Au films irradiated by femtosecond laser pulses at deposited energy densities ranging from 1.21 to 2.85 MJ/kg, i.e., for ε ranging from 5.7 to 13.4 ε_m_ (*17*). The effect of the transient modification of the interatomic interactions, however, can be expected to be minimal under irradiation conditions discussed in the present paper (ε ≤ 1.17 MJ/kg), where the maximum *T*_e_ barely exceeds 17,000 K (fig. S2, tables S2 and S3). According to the analysis provided in (*21*), the transient bond hardening can only be expected when an isochoric electronic excitation of Au brings the electronic temperature above 25,000 K. Moreover, at the time scale of the anomalously slow melting observed in experiments at ε < 0.4 MJ/kg, the electron temperature can be expected to be well equilibrated with the lattice, and the slowdown of the melting process cannot be attributed to any effects related to the electronic excitation.

### Strength of electron-phonon coupling

The initial series of TTM-MD simulations is performed with the electron temperature-dependent electron heat capacity *c*_e_(*T*_e_) and electron-phonon coupling parameter *g*(*T*_e_) calculated based on the electron density of states for Au and accounting for the effect of the thermal excitation of *d* band electrons (*28*, *29*). The consideration of the contribution of the thermal excitation of the *d* band electrons has been shown (*19*, *21*, *28*) to be essential for achieving quantitative agreement with the results of earlier experimental probing of the melting time in thin Au films (*15*, *16*, *21*). The substantially longer melting times reported in (*22*) have been discussed as evidence in favor of a weaker electron temperature dependence and a lower magnitude of the electron-phonon coupling parameter (*22*, *26*, *34*). In particular, the temperature-independent values of *g* linearly increasing with the deposited energy density ε from *g* = 2.2 × 10^16^ W m^−3^ K^−1^ at ε = 0.18 MJ/kg to *g* = 4.9 × 10^16^ W m^−3^ K^−1^ at ε = 1.17 MJ/kg have been suggested to provide an adequate TTM description of the experimental results (*22*).

**Fig. 9.**
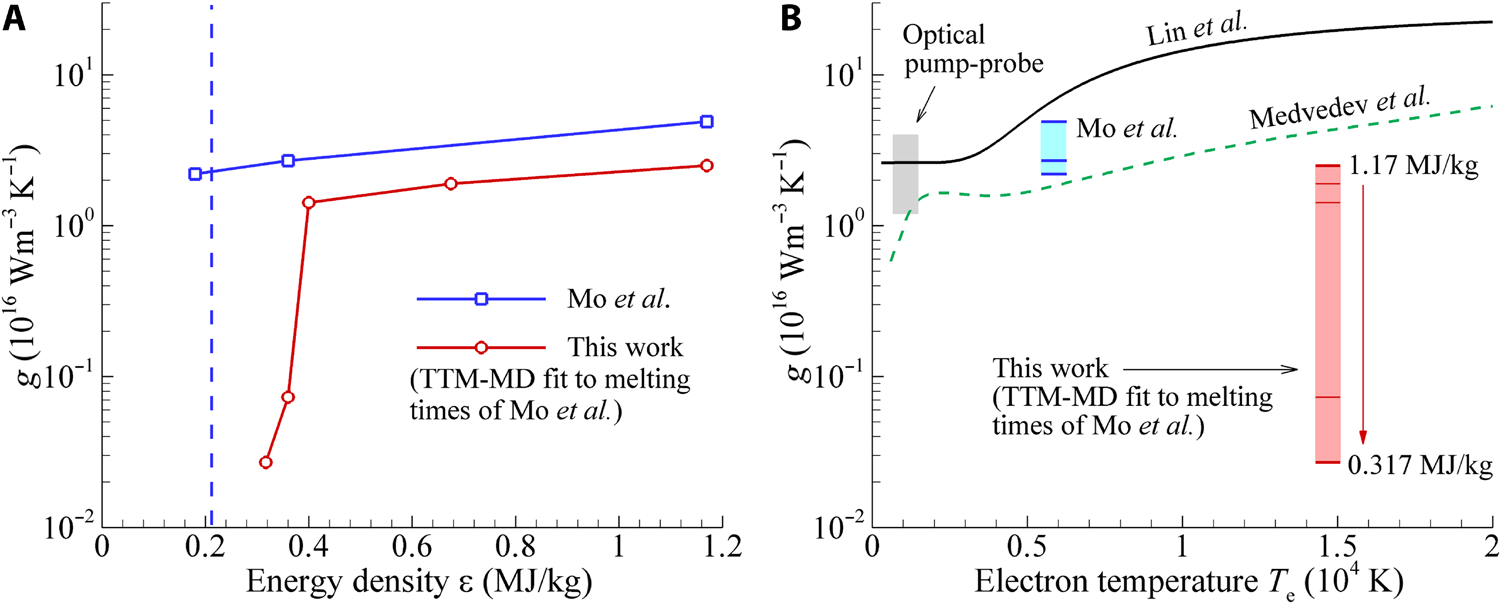
The strength of the electron-phonon coupling in Au predicted theoretically and fitted to the experimental melting times. (**A**) The values of the electron-phonon coupling *g* proposed in (*22*) for the description of the results of electron diffraction probing of the melting process in 35-nm Au films irradiated by 130-fs laser pulses at different deposited energy densities ε (blue line and squares) and calculated in TTM-MD simulations by fitting to the melting times reported in (*22*) (red line and circles). The vertical blue dashed line shows the theoretical threshold for complete melting, ε_m_. (**B**) The electron temperature dependences of the electron-phonon coupling obtained in theoretical studies reported in (*29*) (black curve) and (*34*) (green dashed curve). The gray box shows the range of *g* values evaluated from optical pump-probe measurements (*27*, *44*–*51*). The blue and red levels marked within the boxes of the same color mark the values of *g* shown by the data points in (A). The horizontal positions of the gray, blue, and red boxes are arbitrary and are not related to any particular values of *T*_e_. The maximum values of *T*_e_ and the values of *T*_e_ at the end of the melting process are provided for simulations outlined by the red and blue boxes in tables S2 and S3.

The times of the complete melting obtained in the TTM-MD simulations performed with the temperature-independent *g*(ε) suggested in (*22*) are shown by blue solid circles in Fig. 8. These computational results are closer to the experimental data points, with the agreement being reasonably good in the high-energy regime. The computational predictions, however, are still very far from the anomalously long melting times observed for ε < 0.4 MJ/kg (*54*). To determine the values of *g* that would reproduce the experimental data, we run an additional series of TTM-MD simulations for each energy density in the range from 0.317 to 1.17 MJ/kg for which the experimental data are available. The results are plotted as a function of the deposited energy density in Fig. 9A and are also marked within the red box in Fig. 9B. For comparison, the results of optical pump-probe measurements of *g* performed under conditions of weak laser excitation (*27*, *44*–*51*), when the temperature increase stays within tens to hundreds of K, are shown by the gray box in Fig. 9B. Most of the values obtained by fitting the results of optical probing to the TTM equations are within the range of (2 − 4) × 10^16^ W m^−3^ K^−1^ (*27*, *44*–*49*), although lower values of (1.2 − 1.8) × 10^16^ W m^−3^ K^−1^ have also been reported (*50*, *51*).

The values of *g* obtained by fitting the TTM-MD results to the experimental melting time measured in (*22*) for ε = 0.4, 0.675 and 1.17 MJ/kg are at the lower end of the range obtained with the optical probe but are more than an order of magnitude below this range for ε = 0.317 and 0.36 MJ/kg, where the fitting yields *g* = 0.027 × 10^16^ W m^−3^ K^−1^ and *g* = 0.074 × 10^16^ W m^−3^ K^−1^, respectively. These values of *g* are also far below the theoretical *g*(*T*_e_) curves represented in Fig. 9B by the strongest (*29*) and weakest (*34*) of several dependences reported in the literature (*28*, *29*, *34*–*40*). In the absence of any physical mechanism that could produce such a drastic weakening of the electron-phonon coupling in Au films, we have to conclude that the results of modeling and experiments at energy densities of ε_m_ < ε < 1.7 ε_m_ cannot be reconciled by any reasonable assumption on the strength of the electron-phonon coupling.

### Energy redistribution away from the crystalline parts of the film

The attribution of the slow kinetics of melting at 0.36 MJ/kg (1.7 ε_m_) and 0.317 MJ/kg (1.5 ε*_m_*) to the transition to the regime of heterogeneous melting (*22*) not only contradicts the results of the TTM-MD simulations but also comes into conflict with a simple estimation of the lattice temperature after the electron-phonon equilibration (but before the melting), *T*′ = *T*_m_ + (ε − ε_m_)/*c*_p_(*T*_m_). Using the experimental parameters of Au (*31*), we obtain *T*′ = 1.68 *T*_m_ for ε = 0.36 MJ/kg and *T*′ = 1.48 *T*_m_ for ε = 0.317 MJ/kg. Both of these values are substantially higher than the threshold temperature for the onset of massive homogeneous nucleation, T^*^ ≈ 1.25 *T*_m_, estimated within the classical nucleation theory (see the Supplementary Materials) and confirmed in earlier MD simulations, e.g., (*9*, *13*, *14*, *19*). Since *T*′ > T^*^, one can expect the rapid homogeneous melting to occur within picoseconds after the lattice temperature exceeds T^*^ at these two energy densities. The onset of heterogeneous melting at *T* ≥ *T*_m_ can redistribute the energy away from the crystalline part of the film and reduce the temperature of this part by a maximum of Δ*T* ≈ Δ*H*_m_/*c*_p_(*T*_m_) = 0.29*T*_m_ by the end of the melting process. For this reduction to be substantial, however, the propagation of the two melting fronts through the film and the thermal equilibration between the molten and crystalline parts of the film should take place at the time scale of electron-phonon equilibration, i.e., much faster than the experimentally observed melting times.

Another potential reason for a deviation from the uniform energy distribution throughout the thickness of the film is the contribution of surface scattering to the relaxation of the electrons excited by the laser pulse. It has been discussed, mostly in the context of the size dependence of electron-phonon coupling in metal nanoparticles (*55*–*58*) and thin films (*59*, *60*), that an enhanced energy transfer from the excited electrons to surface vibrational modes may originate from the reduction of the density of conduction band electrons (electron “spill-out” effect), leading to the decreased screening of the electron-ion interactions in the vicinity of the surface. The enhanced coupling near the surfaces may, in principle, produce some nonuniformity of the lattice heating, even though a prompt establishment of a uniform initial electron temperature distribution can be expected for films with thicknesses smaller than the ballistic energy transport (*27*, *32*). Most studies, however, only reveal the effect of the surface on the overall rate of the electron-phonon equilibration for Au nanoparticles with diameters smaller than ~10 nm (*56*–*58*). For Au films, the absence of an apparent size effect is observed in transient thermoreflectance measurements performed for Au films with thicknesses of 10 to 100 nm in (*27*) and 27 to 85 nm in (*61*), while a bulk-like value of *g* = 2.7 × 10^16^ W m^−3^ K^−1^ is obtained by electron diffraction probing of an 8-nm Au film excited up to a maximum *T*_e_ = 2950 K by femtosecond laser irradiation (*62*).

These observations suggest that the preferential heating of surface regions due to the enhanced electron-surface scattering is highly unlikely. Nevertheless, to eliminate any possibility that the slow melting dynamics can be attributed to the locally enhanced electron-phonon coupling, we performed two TTM simulations for ε = 0.36 MJ/kg, where the electron-phonon coupling is artificially increased in 4-Å-wide layers adjacent to the free surfaces of the film up to arbitrary chosen high levels of *g*_s_ = 5 × 10^17^ Wm^−3^K^−1^ and *g*_s_ = 3 × 10^18^ Wm^−3^K^−1^. The results of the simulations, illustrated in fig. S6, demonstrate that the enhanced coupling at the film surfaces leads to the decrease in the maximum temperature in the central part of the film. The melting time, however, is only weakly affected by the artificially increased coupling strength at the surfaces, and the homogeneous melting is still predicted to occur within 10 ps after the laser pulse. We conclude, therefore, that an uneven energy deposition or redistribution through the thickness of the film is very unlikely to be the cause for the prolonged melting times observed in the experiments at energy densities below 0.4 MJ/kg = 1.9 ε_m_.

We also note that the lateral variation of the deposited laser energy within the flat-top laser beam cannot explain the long melting times observed in (*22*), as the beam intensity profile has been carefully characterized in the experiments, and the root mean square intensity variation is found to be less than 5% of the intensity averaged over the whole area probed by the mega electron volt electrons. Moreover, since the characteristic length of the heat diffusion during the time of melting is less than a micrometer, the kinetics of the melting process can only be affected by a submicron lateral variation of the laser energy deposition.

### Electron emission and other channels of energy loss

The energy loss and film charging due to the electron emission can also be eliminated as possible reasons for the anomalously slow melting observed in the experiments. The estimations provided in the Supplementary Materials suggest that both the multiphoton photoelectron emission and thermionic emission are effectively suppressed by the electric field generated by the emitted electrons (*63*, *64*), with the space charge effect reducing the electron emission by several orders of magnitude. In particular, at the threshold for complete melting, the fraction of conduction band electrons emitted from the film is estimated to be below 10^−9^, suggesting that the film charging is unlikely to have any effect on the melting process. The energy loss due to the electron emission also constitutes a negligible fraction of the energy density deposited by the laser pulse.

Last, we note that other slower channels of energy loss following the laser excitation are also unlikely to be responsible for the delayed complete melting of the film. As can be seen in Figs. 2B and 4, the slowest complete melting predicted in the simulation performed at 0.21 MJ/kg, just below the energy density required for the complete melting, occurs on a time scale of about 100 ps. This observation suggests that any channel of energy loss affecting the melting process should have a comparable or shorter time scale. Otherwise, the energy loss would contribute to resolidification rather than melting. In particular, the parts of the Au film adjacent to the sides of the Si wafer windows supporting the film (*22*) may preserve crystallinity due to the heat transfer to the supporting grid and the overall stabilizing effect of the Au-substrate interface. A similar effect was observed in simulations of laser melting of Ag targets covered by a silica glass overlayer (*65*), where crystalline regions adjacent to the overlayer survive the thermal spike caused by the laser excitation. On a longer time scale, however, these crystalline regions do not melt but serve as precursors for resolidification of the transiently melted region.

To summarize, the detailed computational investigation of the femtosecond laser interaction with thin Au films is performed to establish the connections between the strength of electron-phonon coupling, kinetics and channels of thermalization of the deposited laser energy, and the mechanisms of melting occurring under highly nonequilibrium conditions. A particular focus of the computational analysis is on explaining the recent experimental observation of the anomalously slow nanosecond-scale kinetics of melting at energy densities that are 50 to 70% above the threshold for complete melting, ε_m_ (*22*). The results of the simulations reveal a gradual transition from the rapid homogeneous melting within ~5 ps after the laser pulse at the highest energy considered in the simulations, ε=5.5 ε_m_, to slower homogeneous melting within 10 to 20 ps at ε = (1.5 − 1.7)ε_m_, and to the melting process defined by a combined contribution of the homogeneous nucleation of liquid regions in the central part of the film and the propagation of melting fronts from free surfaces of the film in the vicinity of the melting threshold. The dynamic relaxation of laser-induced stresses is playing an increasingly important role in triggering the onset of homogeneous melting as the deposited energy density approaches ε_m_. At ε ≈ ε_m_, the nucleation of liquid regions in the central part of the film takes place upon the unloading of the initial compressive stresses and is conditioned by the interplay of the transient cooling and the depression of the melting temperature by anisotropic stresses produced by the uniaxial expansion of the film. The slowest complete melting of the film is observed just below the theoretical threshold for complete melting, at ε=0.99 ε_m_, where the last crystalline regions are observed to disappear at *T* ≈ 0.99 *T*_m_ by about 130 ps. Note that these small crystalline regions constitute less than 2% of the film, and their contribution can be hardly observed in the diffraction profiles (see Fig. 7C and related discussion above).

The long melting times observed in the experiments performed at energy densities substantially (50 to 70%) above the melting threshold cannot be reconciled with the TTM-MD simulations by any reasonable variation of the electron-phonon coupling parameter. The notion of the dominant contribution of heterogeneous melting at laser energies above the melting threshold, inferred from the experimental observations, is also not confirmed in the simulations, which suggest that the melting front propagation from surfaces of the film is responsible for melting of a relatively small fraction of the material at energy densities above the threshold for complete melting. The apparent lack of theoretical confirmation of the experimental time scales of melting suggests that the use of the values of electron-phonon coupling parameter derived from these experiments for the quantitative verification of theoretical models for the electron temperature dependence of electron-phonon coupling (*22*, *26*, *34*, *39*, *66*) should be done with caution. The intriguing yet unexplained experimental data call for further investigations and highlight the need for well-coordinated experimental and theoretical efforts aimed at fully resolving the mechanisms and kinetics of laser-induced melting.

## MATERIALS AND METHODS

### TTM-MD model

The simulations of laser interaction with Au films are performed with a hybrid atomistic-continuum model that combines the classical MD method with a continuum-level description of the laser excitation and subsequent relaxation of the conduction band electrons provided by a TTM equation for electron temperature *T*_e_ (*67*). The cells in the finite difference discretization used for the solution of the TTM equation for *T*_e_ are related to the corresponding volumes of the MD system, and the local lattice temperature is calculated for each cell from the average kinetic energy of thermal motion of atoms. The energy exchange between the electrons and the lattice is described by coupling terms present in the TTM equation and added to the MD equations of motion. A complete description of the combined TTM-MD model can be found in (*67*).

### Parameters of TTM equation for the electron temperature *T*_e_

The electron temperature dependences of the electron heat capacity *c*_e_(*T*_e_) and the electron-phonon coupling parameter *g*(*T*_e_) used in this study are calculated on the basis of the electron density of states for Au and account for the contribution from the thermal excitation from the electron states below the Fermi level (*28*, *29*). Additional TTM-MD simulations are performed for each value of the deposited energy density ε with several temperature-independent values of electron-phonon coupling parameter, *g*(ε). Among these simulations, one set is performed for values of *g*(ε) suggested in (*22*) based on fitting the experimentally measured melting times to the results of TTM calculations. Another set of simulations is performed for values of *g*(ε) chosen to provide quantitative agreement between the experimental melting times in (*22*) and the predictions of the TTM-MD simulations performed in the present study.

The electron thermal conductivity is described by the Drude model relationship, $k_{e}(T_{e},T_{l})=\frac{1}{3}v_{F}^{2}C_{e}(T_{e})\tau_{e}(T_{e},T_{l})$, where *v*_F_ is Fermi velocity and τ_e_(*T*_e_, *T*_l_) is the total electron scattering time defined by the electron-electron and electron-phonon scattering rates, $1/\tau_{e}=1/\tau_{e-e}+1/\tau_{e-\text{ph}}=AT_{e}^{2}+BT_{l}$. The coefficients *A* = 1.2 × 10^7^ K^−2^s^−1^ and *B* = 1.23 × 10^11^ K^−1^s^−1^ are adopted from (*68*), and several approximations of *k*_e_(*T*_e_, *T*_l_) for Au are compared in (*67*). Note that despite the uniformity of the initial electron temperature distribution, promptly established after the laser excitation (see description of the “Computational setup” section below), the electron heat conduction still plays an important role in the thermal energy redistribution within the film during the melting process. As has been shown in earlier TTM-MD simulations (*10*), the kinetics of heterogeneous melting is defined by the balance between the local temperature drop at the melting front due to the transformation of thermal energy to the latent heat of melting, the electron heat conduction to the melting front, and the electron-phonon coupling. The electron heat conduction and electron-phonon coupling may also play a role in the thermoelastic damping of the laser-induced vibration of the film, as the electron temperature follows the lattice temperature variations induced by the adiabatic expansion and compression of the film with a lag defined by the strength of the electron-phonon coupling (*19*).

### Interatomic potential

The interatomic interaction in the MD part of the model is described by the EAM potential parametrized for Au (*53*). The potential provides an accurate description of the thermodynamic properties of Au relevant to the laser heating and melting processes investigated in the present study. In particular, the equilibrium melting temperature determined in crystal-liquid coexistence simulation is found to be $T_{m}^{\text{EAM}}=1331\;K$, which is within 1% of the experimental value of *T*_m_ = 1337 K (*31*). The energy of the liquid-crystal interface, γ_LC_ = 0.14 J/m^2^, is evaluated for the EAM Au in a series of MD simulations performed for crystalline particles with different radii surrounded by undercooled liquid as described in (*69*). This value is close to the experimental energy of the liquid-crystal interface of 0.132 J/m^2^ (*70*).

The temperature dependence of the lattice heat capacity predicted by the EAM potential for a temperature range from 300 K to $T_{m}^{\text{EAM}}$ can be approximated by a linear dependence *c*_l_(*T*) = *a* + *bT*, where *a* = 23.72 J mol^−1^ K^−1^ and *b* = 4.98 × 10^−3^ J mol^−1^ K^−2^. The contribution of conduction band electrons to the total heat capacity of the material is also accounted for by the TTM-MD model and, in the range of temperatures from 300 K to *T*_m_, can be approximated by a linear dependence, *c*_e_ = γ*T* with γ = 71 J m^−3^ K^−2^ = 0.724 × 10^−3^ J mol^−1^ K^−2^ (*29*). The total heat capacity of the model Au represented by the TTM-MD model, $c_{p}^{\text{TTM}-\text{MD}}(T)=c_{l}(T)+c_{e}(T)$ ranges from 25.43 J mol^−1^ K^−1^ at 300 K to 31.31 J mol^−1^ K^−1^ at $T_{m}^{\text{EAM}}$, which is close to the experimental range of *c*_p_, from 25.3 J mol^−1^ K^−1^ at 300 K to 32.3 J mol^−1^ K^−1^ at *T*_m_ (*31*). The heat of melting predicted by the potential is found to be ${\Delta H}_{m}^{\text{EAM}}=\;12.82$ kJ/mol, which is close to the experimental value of Δ*H*_m_ = 12.72 kJ/mol (*31*). By integrating $c_{p}^{\text{TTM}-\text{MD}}(T)$ from 300 K to $T_{m}^{\text{EAM}}$ and adding ${\Delta H}_{m}^{\text{EAM}}$ (*14*, *19*), the threshold energy density for complete melting predicted by the TTM-MD model of Au is ε_m_ = 0.212 MJ/kg, i.e., the same as the energy density evaluated in Introduction for the experimental parameters of Au.

### Computational setup

The choice of the parameters of the TTM-MD simulations is aimed at reproducing the conditions of experiments reported in (*22*). The simulations are performed for a 35-nm-thick freestanding single-crystal Au film with face-centered cubic (fcc) structure and (100) crystallographic orientation of the two surfaces. The initial dimensions of the TTM-MD domain are 50 nm by 50 nm by 35 nm (corresponds to 4,896,000 atoms), and periodic boundary conditions are imposed in the directions parallel to the free surfaces. The periodic boundaries provide an adequate representation of the conditions, and then the laser spot diameter is sufficiently large, so that the energy redistribution in the lateral directions can be neglected on the time scale considered in the simulations. Before the laser irradiation, the system is thermalized at 300 K for 200 ps.

The irradiation by a 130-fs laser pulse is represented by a source term with a Gaussian temporal profile shifted with respect to the start of the simulation (zero time) by 2.5 × τ_p_, where τ_p_ is the laser pulse duration defined as the full width at half-maximum of the Gaussian profile. The spatial distribution of the energy deposition to the electrons follows the exponential Beer-Lambert law with an effective penetration depth increased to 50 nm to account, in an approximate manner, for the energy transport by nonthermal (ballistic) electrons (*71*, *72*) and further modified (*27*) to ensure that the full amount of energy defined by the absorbed laser fluence is deposited in the Au film. Since the electron temperature equilibration throughout the thickness of the 35-nm film takes place within the first picosecond after the laser pulse (*32*), the effective energy deposition is nearly uniform throughout the thickness of the film.

### Data analysis and calculation of 2D diffraction patterns

The structural analysis of the atomic configurations generated in the simulations is based on the calculation of local order parameter (*67*, *73*) that enables the identification of the crystal and liquid regions in the irradiated films. The value of the order parameter is averaged over neighboring atoms within a cutoff radius of 1.115*a*(1 + *T*_l_ × α_L_), where *a* is a lattice constant at 300 K and α_L_ is the linear coefficient of thermal expansion. A threshold of 0.04 is applied to distinguish between atoms that belong to the solid and liquid phases. The films are considered to be completely molten when the fraction of atoms that belong to the liquid phase exceeds 98%. This choice of the definition of the complete melting is based on the analysis of the diffraction patterns, as discussed in Results.

To enable direct comparison of the computational predictions with the results of ultrafast diffraction probing of the melting process (*15*–*18*, *20*–*22*, *26*), we compute 2D diffraction patterns for atomic configurations generated in the simulations using an approach described in (*14*, *74*). The intensity of the wave scattered at a scattering vector $\vec{Q}$ normalized by the number atoms in the system can be calculated as

$$I(\vec{Q})=\frac{1}{N}\sum_{i=1}^{N}\sum_{j=1}^{N}f_{i}(\vec{Q})f_{j}(\vec{Q})\text{exp}(-i\vec{Q}∙{\vec{r}}_{\mathrm{ij}})$$ (1)

where $f_{i}(\vec{Q})$ are atomic scattering factors, ${\vec{r}}_{\mathrm{ij}}$ are vectors between atoms *i* and *j*, and the summation is over the total number of atoms *N*. For a monoatomic system, $f_{i}(\vec{Q})=f_{j}(\vec{Q})$, and this equation can be rewritten as

$$I(\vec{Q})={|f(\vec{Q})|}^{2}[1+\frac{1}{N}\sum_{\begin{array}{c} i,j=1\\ i\neq j \end{array}}^{N}\text{cos}(\vec{Q}∙{\vec{r}}_{\mathrm{ij}})]$$ (2)

The dot product of the scattering vector and interatomic vector can be rewritten as $\vec{Q}∙{\vec{r}}_{\mathrm{ij}}=Q{\vec{e}}_{Q}∙{\vec{r}}_{\mathrm{ij}}$, where *Q* is the magnitude of the scattering vector $\vec{Q}$ and ${\vec{e}}_{Q}$ is a unit vector parallel to $\vec{Q}$. For the high-energy electron diffraction (3.2 MeV) (*22*), the Ewald sphere can be considered to be flat over the region of reciprocal space of interest. Thus, to reproduce experimental results, we perform calculations for a (*Q_x_*, *Q_v_*, 0) slice parallel to the surface of the film, i.e., assuming that both $\vec{Q}$ and ${\vec{e}}_{Q}$ are 2D. The unit vector ${\vec{e}}_{Q}$ can then be described by a single parameter which is an angle θ with respect to the *Q_x_* axis. In the calculations, we use a discrete set of ${\vec{e}}_{Q}$ vectors, with an angular resolution of 1.0°. These vectors are defined by the discrete set of angles θ*_n_*. To improve the computational efficiency of the calculation, we constructed a 2D histogram *h*(θ_n_, *r*_k_) that contains the number of projections of ${\vec{r}}_{\mathrm{ij}}$ on ${\vec{e}}_{Q}$ defined by θ_n_ that falls within (*r*_*k*,_ *r_k_* +Δ*r*) range, where Δ*r* = 0.04 Å is a histogram bin size. The calculations account for the pairs of atoms that are within a cutoff distance of *R*_max_ = 31.4 Å from each other, which corresponds to *Q*_min_ *=* 0.2 Å^−1^.

To mitigate the finite size effect, we include the contribution of each projection of ${\vec{r}}_{\mathrm{ij}}$ on ${\vec{e}}_{Q}$ to the histogram with a weight defined by the damping function *W*(*r_ij_*) = sin (π*r_ij_*/*R*_max_)/(π*r_ij_*/*R*_max_) (*14*). Similar to the commonly used modification of 1D radial distribution function, e.g., (*75*), the histogram *h*(θ, *r_k_*) is further modified by subtracting $M(r_{k})=\frac{4R_{\text{max}}^{2}n_{0}\Delta r}{\pi \;}[1+\text{cos}(\frac{\pi r_{k}}{R_{\text{max}}})]$, where *n*_0_ is the average number density of the system.

Using the approach described above, the scattering intensity distribution in polar coordinates (*Q*, θ) as can be expressed as

$$I(\vec{Q})=I(Q,\theta )={|f(Q)|}^{2}(1+\sum_{k=1}^{n_{\text{bins}}}\{h(\theta_{n},r_{k})/N-M(r_{k})\}\times \text{cos}(Qr_{k}))$$ (3)

where summation is over all the radial histogram bins, *n*_bins_. The atomic scattering factor is calculated using a parametrization ${|f(Q)|}^{2}={\left(\sum_{i=1}^{5}a_{i}\text{exp}(-b_{i}Q^{2})\right)}^{2}$, where parameters *a_i_* and *b_i_* for are taken from (*76*). The maximum *Q* value is chosen to be 8.0 Å^−1^, similar to that used in the experiments (*22*), and the values of *Q* are varied with a step of 0.0875 Å^−1^ in the calculation of the 2D diffraction patterns. The computational code implementing the computational method described above is available at (*77*).

## Supplementary Materials

### This PDF file includes:

Supplementary Materials Figs. S1 to S7 References
