# Supplementary material for: Kinetics of laser-induced melting of thin gold film: How slow can it get?
Source: Sci Adv. 2022 Sep 21;8(38):eabo2621. doi: 10.1126/sciadv.abo2621 (PMC9491712; doi:10.1126/sciadv.abo2621)
Supplement: Supplementary file 1 — Supplementary Materials Figs. S1 to S7 References [file sciadv.abo2621_sm.pdf]

Science Advances
AAAS

Supplementary Materials for

Kinetics of laser-induced melting of thin gold film: How slow can it get?

Mikhail I. Arefev, Maxim V. Shugaev, Leonid V. Zhigilei

Corresponding author: Leonid V. Zhigilei, lz2n@virginia.edu

Sci. Adv. **8**, eabo2621 (2022)
DOI: 10.1126/sciadv.abo2621

This PDF file includes:

Supplementary Materials
Figs. S1 to S7
References

Supplementary Material 1: The limit of superheating of Au from the classical nucleation theory, figure S1.

The limit of superheating for the onset of massive homogeneous nucleation of liquid regions inside a superheated Au crystal can be estimated based on the classical nucleation theory (*32*, *78*). The basic steps of the analysis of the limit of superheating are illustrated in fig. S1, where all plots are calculated using the experimental parameters of Au (*31*, *79*). The difference in the volumetric free energy between the solid and liquid phases, ΔG_{sl}, is increasing with the level of superheating above the melting temperature, as can be seen in fig. S1A. Using this temperature dependence of ΔG_{sl} and the solid-liquid interfacial free energy of γ_{sl} = 132×10^{-3} Jm^{-2} (*70,79*), the change in the Gibbs free energy ΔG due to the formation of a spherical nucleus with a radius r can be calculated as a function of r, fig. S1B. For any given level of superheating, the critical radius r_c that corresponds to the maximum of $\Delta G(r)$ and the corresponding energy barrier $\Delta G(r_c)$ can be calculated, as shown in fig. S1B.

The supercritical nuclei with $r > r_c$ will grow spontaneously, and the rate of the nucleation can be expressed as $R = R_0 \times exp(-\Delta G(r_c)/k_B T)$, where k_B is the Boltzmann constant and R_0 is a pre-factor weakly dependent on temperature. The nucleation rate required to completely melt a crystal within a melting time of τ_m can be estimated as $R \approx (2v\tau_m)^{-3}\tau_m^{-1}$, where v is the velocity of the propagation of crystal-liquid interfaces generated by the nucleation. According to the results of MD simulations, the duration of the homogeneous melting at the limit of superheating is $\tau_m \approx$ 10 ps (*13*), the maximum velocity of the melting front in metals is $v \approx$ 500 m/s (*10*), and the limit of superheating at the onset of rapid homogeneous nucleation for Au is $T^* \approx 1.25T_m$ (*14*). The values of τ_m and v yield $R \approx 10^{35}$ s^{-1}m^{-3} as an estimate of the nucleation rate at the limit of superheating, while $T^* = 1.25T_m$ corresponds to the volumetric free energy difference of $\Delta G_{sl} \approx$ 3×10^{8} Jm^{-3} (Fig. S1A) and the nucleation barrier of $\Delta G(r_c) \approx 17.4\ k_B T^* \approx$ 2.5 eV (fig. S1B).

The prefactor $R_0 \approx 4\times10^{42}$ s^{-1}m^{-3} that reconciles the above estimations of R and $\Delta G(r_c)$ at T^* is of the same order of magnitude as the theoretical estimation for the nucleation in condensed systems provided in Ref. (*78*), $R_0 \approx k_B T/hV_a$, where V_a is the atomic volume, and h is the Planck constant. The conclusion that the threshold-like onset of the massive homogeneous nucleation observed in MD simulations is consistent, at a semi-quantitative level, with the estimations based on the classical nucleation theory is remarkable. Indeed, the physical picture of the nucleation and

growth of spherical liquid regions cannot be expected to be valid up to T^*, when the critical radius r_c decreases down to less than a nanometer, Fig. S1B, and the distance the melting front propagates during the melting time of $\tau_m = 10$ ps is less than 5 nm. Nevertheless, the kinetics of a more complex melting process revealed in the MD simulations, where a simultaneous formation of a large number of interconnected small liquid regions within the entire superheated volume is observed (*10*, *13*, *14*), is found to still be reasonably well described by the classical nucleation theory.

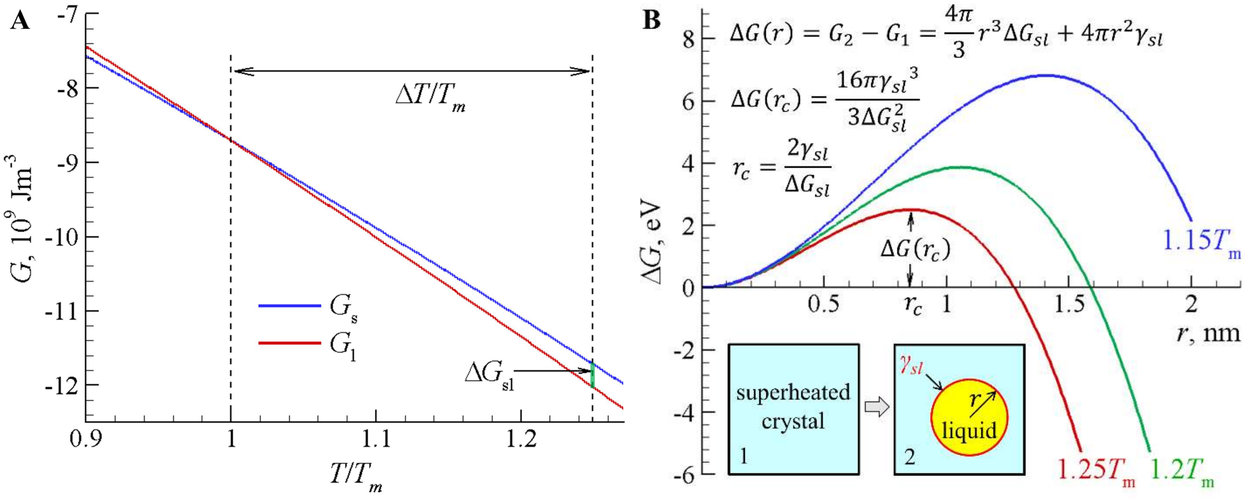

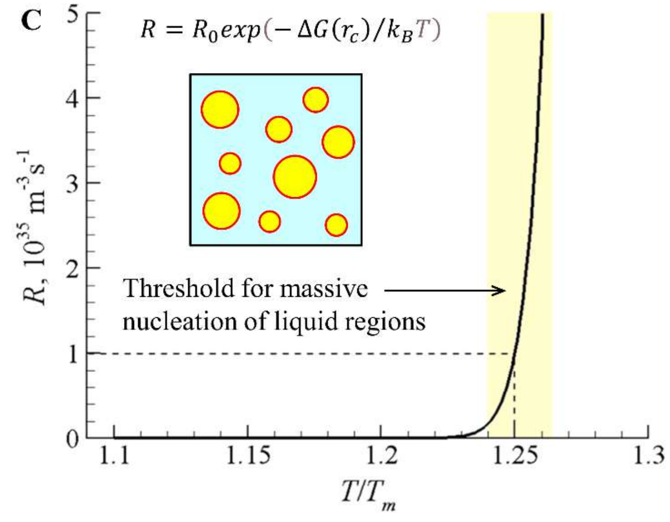

Figure S1. Three steps in the analysis of melting within the classical nucleation theory. (**A**) calculation of the volumetric free energy change ΔG_{sl} associated with melting, (**B**) free energy change $\Delta G(r)$ due to the appearance of a spherical liquid region of radius r inside a superheated crystal, and (**C**) the temperature dependence of the nucleation rate R. All calculations are done with experimental thermodynamic properties of Au (*31*, *79*).

Supplementary Material 2: Evolution of lattice and electron temperatures at ε = 1.17 MJ/kg ($5.5\varepsilon_m$), figure S2.

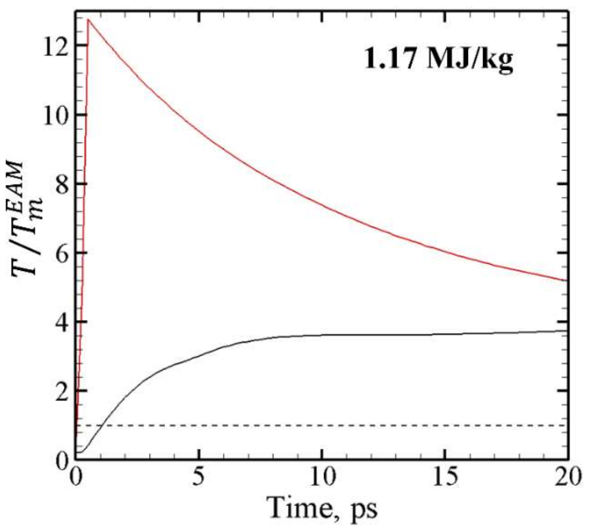

Figure S2. Evolution of lattice and electron temperatures at ε = 1.17 MJ/kg ($5.5\varepsilon_m$). Average lattice (black) and electron (red) temperatures vs. time in a 35 nm Au film irradiated by a 130-fs laser pulse at an absorbed energy density of 1.17 MJ/kg ($5.5\varepsilon_m$). The temperatures are normalized by the equilibrium melting temperature at zero pressure of the model material, $T_m^{EAM} = 1331$ K.

Supplementary Material 3: Temperature, pressure and phase state contour plots obtained in a TTM-MD simulation performed at ε = 0.21 MJ/kg (0.99ε_m) with g = 2.3×10^{16} $Wm^{-3}K^{-1}$, figure S3.

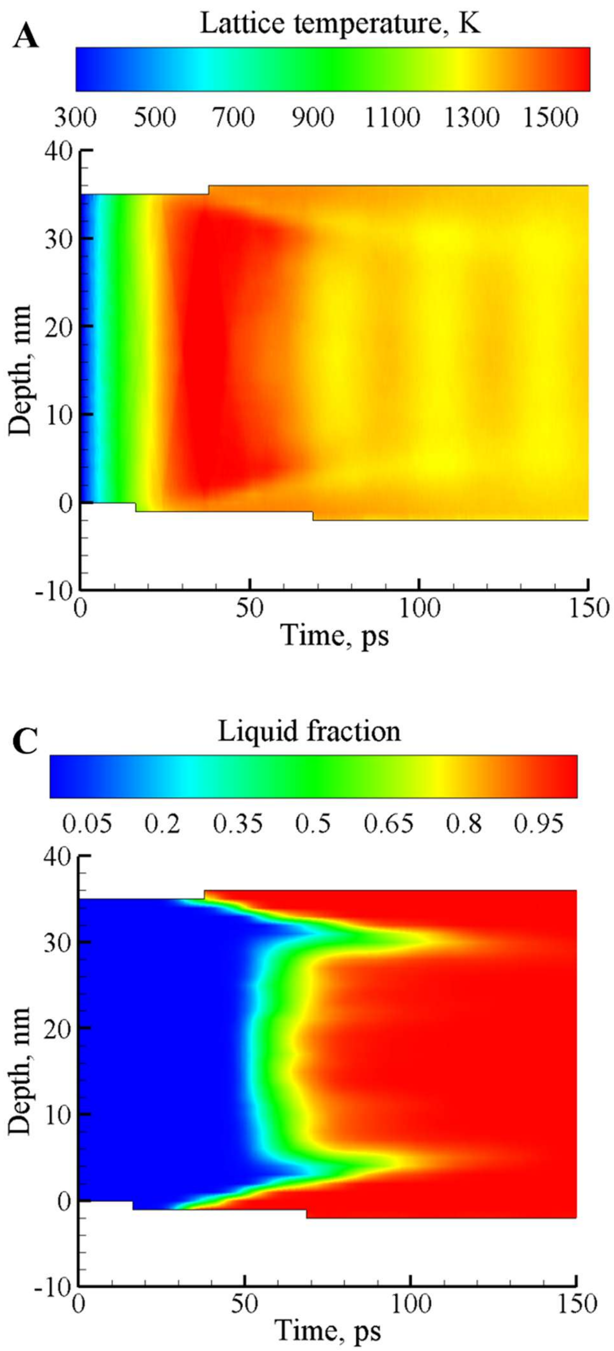

Figure S3. The evolution of temperature, pressure and phase state in a 35 nm Au film irradiated by a 130-fs laser pulse at an absorbed energy density of 0.21 MJ/kg. The contour plots show the spatial and temporal evolution of lattice temperature (**A**), pressure (**B**), and fraction of atoms in the liquid phase (**C**) predicted in a TTM-MD simulation performed with a constant value of electron-phonon coupling parameter, g = 2.3×10^{16} $Wm^{-3}K^{-1}$ obtained from the linear $g(\varepsilon)$ dependence suggested in Ref. (*22*).

Supplementary Material 4: Snapshots of atomic configurations and the evolution of subcritical and supercritical crystalline regions in a TTM-MD simulation performed at ε = 0.20 MJ/kg ($0.94\varepsilon_m$), figures S4 and S5.

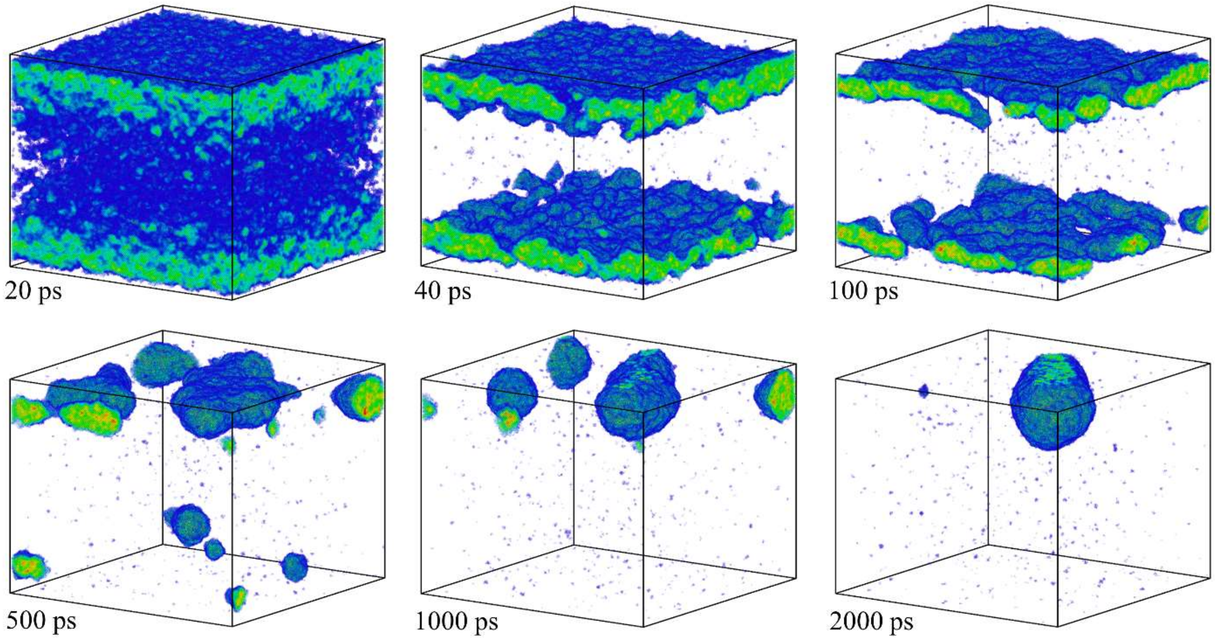

Figure S4. **Atomic snapshots illustrating the melting process of a 35 nm Au film irradiated by a 130-fs laser pulse at an absorbed laser energy density of 0.2 MJ/kg ($0.94\varepsilon_m$).** The snapshots are from a TTM-MD simulation performed with $g(T_e)$ from Ref. (*29*). The atoms that belong to the liquid phase are blanked, and the remaining atoms are colored according to the local order parameter, with color variation from blue to red for the local order parameter ranging from 0.04 to 1.

At an absorbed energy density of 0.2 MJ/kg, which is about 6% below the threshold for complete melting, the total number of atoms in the regions that remain crystalline decreases until about 2 ns, and then starts to increase, fig. S5A. This observation can be explained by the disintegration of the crystalline layers into separate regions during the first several hundreds of picoseconds after the laser pulse, fig. S4. The crystalline regions with effective diameters below $D^* = (4\gamma_{LC}T_m)/(\Delta H_m \Delta T)$ are thermodynamically unstable at $T = T_m - \Delta T$ (*19*) and disappear even though $T < T_m$. By the time of 1 ns, only three largest crystalline regions with effective diameters

of 9.8, 10.1, and 18.4 nm are present in the molten film. Note that some of the crystalline regions are split into two or three parts in the images shown in fig. S4 due the periodic boundary conditions.

The continuous crystalline regions are identified using cluster analysis implemented in OVITO (*80*) and the evolution of their sized is tracked from 1 ns to 2.5 ns with 20 ps intervals, as shown in fig. S5B. The temperature established in the film by 1 ns is about $0.96T_m$, and the undercooling of $\Delta T = 0.04T_m$ corresponds to $D^* = 11.2$ nm. The temperature and the corresponding value of D^* do not change significantly during the slow evolution of the phase composition of the film after 1 ns. As expected from the analysis of the thermodynamic stability of the crystalline regions, two smaller regions with $D < D^*$ disappear by about 2 ns, while the largest region with $D > D^*$ slowly grows in size, fig. S5B.

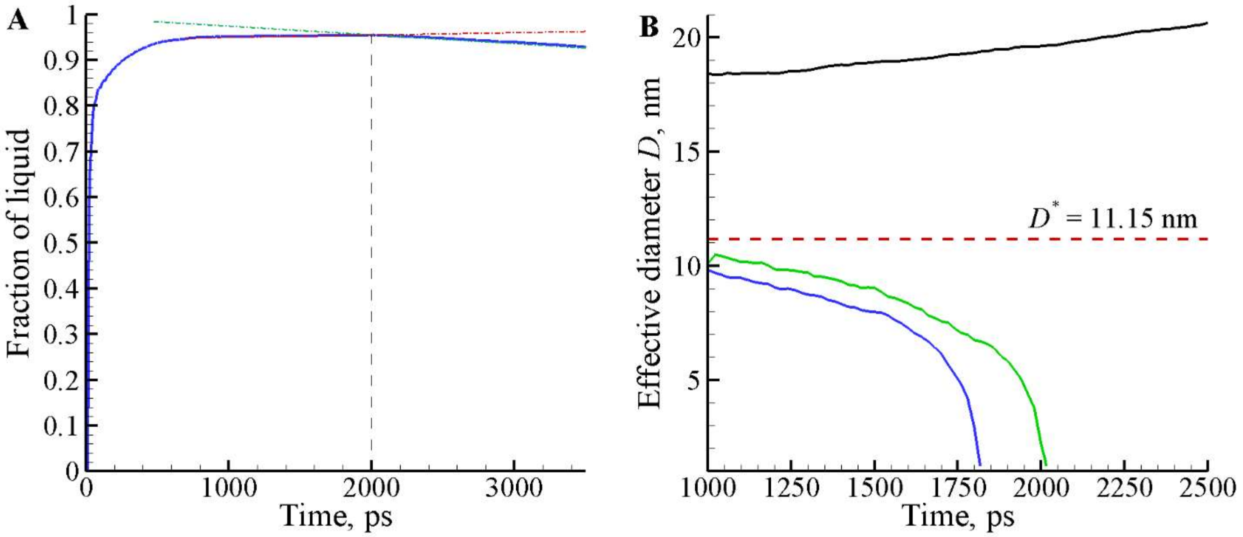

Figure S5. **The long-term evolution of subcritical and supercritical crystalline regions in the simulation of melting of a 35 nm Au film irradiated by a 130-fs laser pulse at an absorbed energy density of 0.2 MJ/kg ($0.94\varepsilon_m$)**. The evolution of the fraction of atoms in the liquid phase is shown in (**A**), and the effective diameters of the three largest crystalline regions that remain in the film at 1 ns are plotted in (**B**). The vertical dashed line in (**A**) marks a time of 2 ns, when the melting turns into crystallization, as indicated by the green and red dash-dotted lines showing the approximate slopes of the melting curve before and after 2 ns, respectively. The horizontal dashed line in (**B**) marks the value of the theoretical critical diameter D^* calculated for the average temperature of the film, $0.96T_m$.

Supplementary Material 5: Tables of the melting times, electron-phonon coupling parameters, and electron temperature predicted in the TTM-MD simulations and obtained from the ultrafast electron diffraction experiments (*22*), tables S1, S2, and S3.

Table S1. The values of the melting times and electron-phonon coupling parameter predicted in the TTM-MD simulations and obtained from the ultrafast electron diffraction experiments (*22*).

Notation: ε – energy density deposited by the laser pulse;

ε_m – theoretical threshold energy density for complete melting of the Au film;

τ_{start} and τ_{end} – the times of the start and end of the melting process, defined as times when the fraction of atoms that belong to the liquid phase reaches the levels of 10% and 98%, respectively;

τ_{end}^{exp} - time of melting reported in Ref. (*22*).

ε MJ/kg	$\varepsilon/\varepsilon_m$	TTM-MD with $g(T_e)$ (*29*)		TTM-MD with $g(\varepsilon)$			Exp. (*22*)
		τ_{start}, ps	τ_{end}, ps	g from (*22*) 10^{16} Wm^{-3}K^{-1}	τ_{end}, ps	g fitted to τ_{end}^{exp} 10^{16} Wm^{-3}K^{-1}	τ_{end}^{exp}, ps
0.18	0.85	43	-	2.20			
0.19	0.90	18.5	-	2.23			
0.195	0.92	17.5	-	2.24			
0.20	0.94	16.7	-	2.26			
0.21	0.99	15.5	56	2.29	115.4		
0.22	1.04	15	35	2.31	55.8		
0.23	1.08	13.75	21	2.34			
0.25	1.18	12.5	17	2.39			
0.30	1.42	9.75	13.75	2.53	34.1		
0.317	1.50	8.8	12.9	2.58	32.1	0.027	2500
0.36	1.70	7.5	11.75	2.70	26.3	0.074	800
0.40	1.89	6.5	11	2.80	23.6	1.44	45
0.50	2.36	5.2	9.4	3.08	19.4		
0.675	3.18	4.0	7.6	3.55	16.6	1.9	25
1.17	5.52	2.8	5.0	4.90	11.2	2.8	15

Table S2. The maximum values of T_e and the values of T_e at the end of the melting process in simulations performed with constant values of g fitted to the time of melting reported in Ref. (*22*), τ_{end}^{exp}. The values of g are outlined by the red box in Fig. 9.

ε, MJ/kg	g fitted to τ_{end}^{exp}, 10^{16} Wm^{-3}K^{-1}	Maximum T_e, 10^3·K	T_e at the end of melting, 10^3·K
0.317	0.027	9.63	5.81
0.36	0.074	10.15	6.85
0.4	1.44	10.59	7.16
0.675	1.9	13.31	10.78
1.17	2.8	17.18	14.67

Table S3. The maximum values of T_e and the values of T_e at the end of the melting process in simulations performed using constant values of g suggested in Ref. (*22*). The values of g are outlined by the blue box in Fig. 9.

ε, MJ/kg	g from (*22*), 10^{16} Wm^{-3}K^{-1}	Maximum T_e, 10^3·K	T_e at the end of melting, 10^3·K
0.317	2.58	9.56	5.35
0.36	2.70	10.08	6.35
0.4	2.80	10.57	7.07
0.675	3.55	13.28	10.41
1.17	4.90	17.16	14.35

Supplementary Material 6: The results of TTM simulations with artificially increased electron-phonon coupling at surfaces of the film, figure S6.

The hypothesis that the slow melting dynamics can be attributed to the local enhancement of the strength of electron-phonon coupling due to the surface scattering is checked and discarded by performing two TTM simulations where the electron-phonon coupling is artificially increased by up to two orders of magnitude in 4-Å-wide layers adjacent to the free surfaces of the film. The TTM simulations are performed with the same parameters of the equation for the electron temperature, *i.e.*, $c_e(T_e)$, $g(T_e)$, and $k_e(T_e, T_l)$, as those described in Materials and Methods in this article, and with the experimental temperature dependence of the lattice heat capacity, $c_l(T_l)$ (*31*). The possibility of the homogeneous melting is not included in the simulations, but can be expected to occur within picoseconds after the temperature exceeds the limit of superheating, $T^* \approx 1.25T_m$, as discussed in Supplementary Material 1. The description of the kinetics of melting front propagation (heterogeneous melting) is incorporated into the model as described in Ref. (*81*). A linear dependence of the melting front propagation velocity on the superheating, $v(T_l) = \mu(T_l - T_m)$, where $\mu = 0.39$ $\mathrm{mK^{-1}s^{-1}}$ (*82*), is assumed in the simulations.

The temperature profiles predicted for the center of the film in the simulations performed for ε = 0.36 MJ/kg with two values of electron-phonon coupling parameter assumed in the surface layers, $g_s = 5 \times 10^{17}\ Wm^{-3}K^{-1}$ and $g_s = 3 \times 10^{18}\ Wm^{-3}K^{-1}$, are shown in Fig. S6. One can see that the enhancement of coupling at the two surfaces is indeed decreasing the heating rate in the center of the film due to a larger amount of energy deposited at the periphery. The enhanced coupling at the film surfaces, however, has a relatively weak effect on the time of melting. Similar to the TTM-MD simulation (Figs. 1A and 2A), the homogeneous melting is still predicted to occur by 10 ps after the laser pulse. If we neglect the possibility of the homogeneous melting, the two melting fronts would meet each other in the middle of the film at 68 to 79 ps, *i.e.*, completing the melting much earlier than the experimentally observed melting time of 800 ps (*22*).

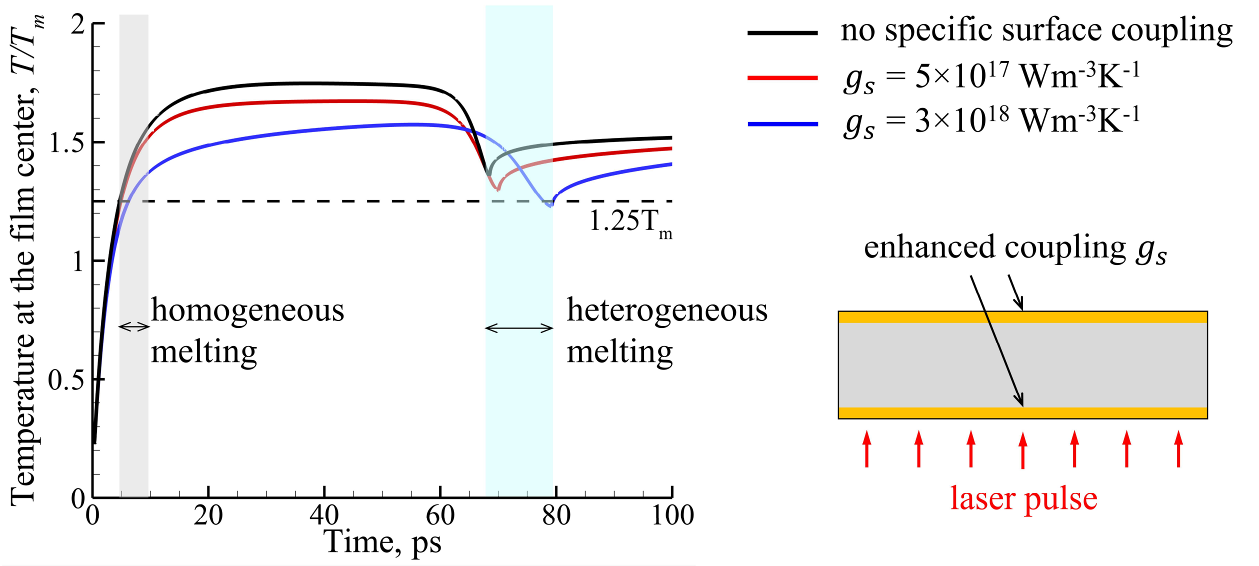

Figure S6. The results of the computational analysis of the effect of the increased electron-phonon coupling at surfaces of a 35 nm Au film irradiated by a 130-fs laser pulse. The plots show the evolution of temperature in the center of the film irradiated at an absorbed energy density of ε = 0.36 MJ/kg, as predicted in TTM simulations performed with and without an artificially enhanced values of electron-phonon coupling in the surface regions. The gray and blue regions mark the timescales of the homogeneous melting at the limit of superheating and the heterogeneous melting under conditions when the homogeneous melting is neglected.

Supplementary Material 7: Evaluation of the effect of thermionic emission and multiphoton photoelectron emission on laser-induced melting.

The electron emission from the free surfaces of the Au film irradiated by the femtosecond laser pulse is a process that is not included in the TTM-MD model used in the simulations of laser-induced melting reported in the present paper. The assumption of the negligible role the electron emission plays in the melting process is verified by performing an order-of-magnitude estimation of the number of ejected electrons under irradiation conditions considered in the present study.

The estimation is performed by solving the TTM equations coupled with the description of the multiphoton photoelectron emission and thermionic emission. The large radius of the flat-top laser beam (R_0 = 210 μm) and the small thickness of the Au film (h = 35 nm) used in the experimental probing of the melting process (*22*) makes it possible to neglect the spatial dependence for electron and lattice temperatures in the model. The TTM equations in this case take the following form:

$$C_e(T_e)\frac{\partial T_e}{\partial t} = -g(T_e)(T_e - T_l) + \Sigma_{abs}(t)/h - Q_{loss}(T_e), \quad \text{(S1)}$$

$$C_l\frac{\partial T_l}{\partial t} = g(T_e)(T_e - T_l), \quad \text{(S2)}$$

where the electron temperature dependence of the electron heat capacity and electron-phonon coupling, $C_e(T_e)$ and $g(T_e)$, are the same as those described in Materials and Methods in the article. Since the goal of the calculations is to obtain a rough order-of-magnitude estimate of the electron emission, we neglect the temperature dependence of the lattice heat capacity and use a room temperature value of $C_l = 2.475 \times 10^6$ $\mathrm{Jm^{-3}K^{-1}}$ (*31*). The source term $\Sigma_{abs}(t)$ represents the laser energy absorbed by the film per unit area and unit time. It is related to the incident laser intensity $\Sigma(t)$ as $\Sigma_{abs}(t) = (1 - R_\omega)\Sigma(t)$, where R_ω is the reflectivity at a given laser wavelength. The laser intensity has a temporal Gaussian profile with the full width at half maximum equal to the laser pulse duration τ_p = 130 fs. To ensure the complete laser energy deposition, the laser pulse is shifted by 2.5× τ_p with respect to the start of the simulation.

The description of the total electron emission current density from each of the two surfaces of the film accounts for the contributions from the multiphoton and thermionic emission (*83*). At the experimental wavelength of 400 nm, the photon energy ($\hbar\omega$ = 3.1 eV) is less than the work function of bulk gold, ϕ = 4.8 eV (*84*), so two- and three-photon photoemission should have the

most significant influence. The two- and three-photon generated electron current density is described by the model based on the Fowler-DuBridge theory (*83*, *85*):

$$J_{2p} = a_{2p} A_0 \left(\left(\frac{e}{\hbar\omega} \right) (1 - R_\omega) \right)^2 T_e^2 F(X_{2p}) \Sigma(t)^2, \tag{S3}$$

$$J_{3p} = a_{3p} A_0 \left(\left(\frac{e}{\hbar\omega} \right) (1 - R_\omega) \right)^3 T_e^2 F(X_{3p}) \Sigma(t)^3, \tag{S4}$$

where $F(x)$ is the Fowler function (*85*, *86*), R_ω is the reflectivity at a given laser wavelength, $A_0 = 120\ \mathrm{A\ cm^{-2}K^{-2}}$ is the Richardson coefficient, e is the elementary charge, a_{2p} and a_{3p} are material-dependent coefficients, which correspond to the two- and three-photon emission, respectively, $X_{2p} = (2\hbar\omega - \tilde{\phi} + \mu(T_e) - E_f)/k_B T_e$, $X_{3p} = (3\hbar\omega - \tilde{\phi} + \mu(T_e) - E_f)/k_B T_e$. Here, $\mu(T_e) - E_f$ is the chemical potential that accounts for the significant change in the effective Fermi energy level caused by the thermal excitation of the *d*-band electrons at high T_e (*29*).

The coefficients a_{2p} and a_{3p} are calculated using relationships between the electron current density and the intensity of incident nanosecond laser pulses determined in experiments reported in Ref. (*84*). The proportionality coefficients for two- and three- photon emission obtained in the experiments are $2.4 \times 10^{-3}\ (\mathrm{A\ cm^{-2}})/(\mathrm{MW\ cm^{-2}})^2$ and $1 \times 10^{-7} (\mathrm{A\ cm^{-2}})/(\mathrm{MW\ cm^{-2}})^3$ at laser wavelengths of 347.2 and 694.3 nm, respectively. Based on values of reflectivity R_ω at these wavelengths, 0.3853 and 0.9695 (*87*), and using Eqs. (S3) and (S4), we obtain the coefficients $a_{2p} = 1.827 \times 10^{-32}\ (\mathrm{m^2/A})^2$ and $a_{3p} = 7.974 \times 10^{-42}\ (\mathrm{m^2/A})^3$. The term $\tilde{\phi}$ is a work function modified to account for the space charge effect as discussed below.

The thermionic emission is described by the Richardson-Dushman equation based on the free electron gas model (*63*):

$$J_t = A_0 T_e^2 \exp\left(-\frac{\tilde{\Phi} - (\mu(T_e) - E_f)}{k_B T_e} \right). \tag{S5}$$

The total electron emission from each of the two surfaces of the film is $J_S = J_{2p} + J_{3p} + J_t$. Since the optical penetration depth is comparable with the film thickness and the film is uniformly heated by the laser pulse, the emission from the front and back surfaces of the film can be assumed to be approximately the same. Thus, the total electron current density calculated in the simulation is $J_{Tot} = 2J_S$. The corresponding numbers of emitted electrons per unit volume of the Au film per

unit time are calculated from the continuity equations, $dn_e^{2p}/dt = 2J_{2p}e^{-1}h^{-1}$, $dn_e^{3p}/dt = 2J_{3p}e^{-1}h^{-1}$, $dn_e^{t}/dt = 2J_t e^{-1}h^{-1}$, and $dn_e^{Tot}/dt = J_{Tot}e^{-1}h^{-1}$.

As discussed in (*88*), each thermionically emitted electron on average carries an energy of $\tilde{\phi} + E_f - \mu(T_e) + k_B T_e$. Thus, the energy loss due to thermionic emission is

$$Q_{loss}^{t}(T_e) = A_0 e^{-1} T_e^2 \left(\tilde{\phi} - (\mu(T_e) - E_f) \ + k_B T_e\right) \exp\left[-\frac{\tilde{\phi} - (\mu(T_e) - E_f)}{k_B T_e}\right], \quad \text{(S6)}$$

and the total energy loss term present in the TTM equation for T_e can be written as

$$Q_{loss}(T_e) = Q_{loss}^{t}(T_e)/h + 2\hbar\omega \times \partial n_e^{2p}/\partial t + 3\hbar\omega \times \partial n_e^{3p}/\partial t, \quad \text{(S7)}$$

The space charge generated by emitted electrons is known to significantly affect the number of escaped electrons (*63*, *64*). To account for this effect, we follow an approximate treatment of the extra energy barrier for the emission of a new electron produced by the cloud of already escaped electrons suggested in Ref. (*63*). Within this treatment, the extra energy barrier is introduced through the modification of the work function,

$$\tilde{\phi} = \phi + a \frac{e^2 n_e^{Tot}(t) \times S \times h}{2\varepsilon_0 R_0}, \quad \text{(S8)}$$

where $n_e^{Tot}(t)$ is the cumulative number of electrons per unit volume emitted by time *t*, ε_0 is the permittivity of vacuum, and $S = \pi R_0^2$, is the area of the laser spot. Coefficient a describes the geometry of the electronic cloud and is assumed here to be 1.7, which corresponds to a thin disk (*63*). The factor of 2 in the denominator of Eq. (S8) is related to the fact that only half of the ejected electrons, $n_e^{Tot}(t)$, contributes to the extra energy barrier at each surface of the film. While a more accurate treatment of the evolution of the cloud of emitted electrons above the target surface is possible in particle-in-cell (PIC) simulations (*64*), the analytical approximation described above has been shown to provide a good quantitative description of the electron emission in experiments performed for several metals (*63*, *68*). The neglect of the space charge effect has been demonstrated to result in an overestimation of the number of electrons emitted through both the thermionic and multiphoton mechanisms by several orders of magnitude (*63*, *64*).

To evaluate the possible effect of the multiphoton and thermionic emission on the melting behavior analyzed in the present paper, we apply the model described above to estimate the number of electrons emitted from a 35-nm-thick Au film irradiated by a 130 fs at an energy density that

corresponds to the threshold for complete melting, $\varepsilon_m = 0.212\,\mathrm{MJ/kg}$. The initial density of conduction band electrons, $n_e^0 = 5.91 \times 10^{28}\mathrm{m}^{-3}$, is calculated by assuming one conducting electron per Au atom.

The results of the simulation are illustrated by Fig. S7. The rapid build-up (within 0.4 ps) of the space charge due to photoemission leads to the buildup of the repulsive electrostatic potential up to 15.3 V, the level similar to that predicted in PIC simulations performed for Cu (*64*). This potential suppresses further emission, with the electron emission current density due to multiphoton emission exhibiting a pronounced dip at ~ 0.125 ps and a partial transient recovery produced by the competition between an increase in the effective work-function $\tilde{\phi}$ and a simultaneous increase in the electron temperature, fig. S7B. Since the model does not include any sinks for the emitted electrons, the final fraction of emitted electrons is mostly defined by the two- and three- photon photoemission rather than a longer-term thermionic contribution. The total number of the emitted electrons does not exceed $10^{-7}\%$ of the total number of conduction band electrons, fig. S7C. Such a small fraction of emitted electrons is unlikely to have any effect on the magnitude of the electron-phonon coupling or affect lattice stability against melting. The total energy loss due to electron emission is found to be on the order of $\sim 10^{-3}\mathrm{J/kg}$, and the effect of this energy loss on the electron temperature is negligible.

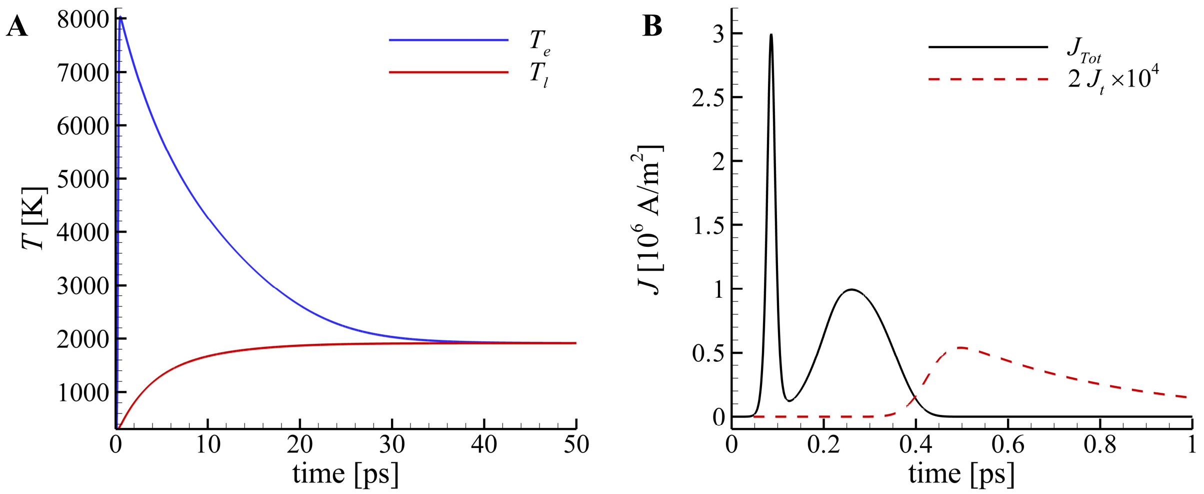

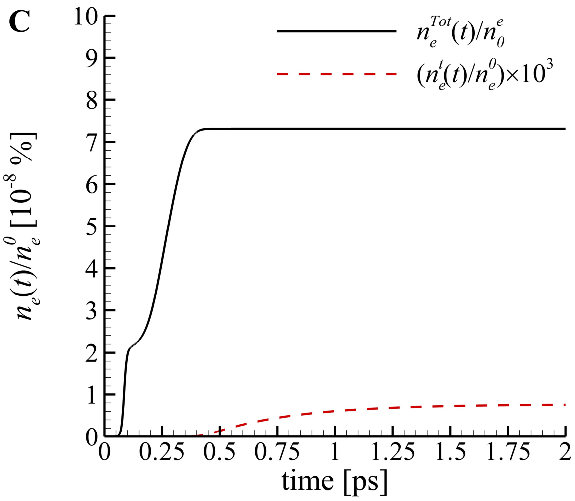

Figure S7. The results of the TTM-based estimation of the electron emission from a 35 nm Au film irradiated by a 130-fs laser pulse. The simulation is performed at an energy density that corresponds to the threshold for the complete melting of the film. The evolution of electron (black line) and lattice (red line) temperatures is shown in (**A**). The total electron current density (black line) and the thermionic contribution (red dashed line) are shown in (**B**). The total number of emitted electrons (black line) and fraction of electrons emitted due to the thermionic emission only (red dashed line) are shown in (**C**). The thermionic current density and the corresponding number of emitted electrons are multiplied by $\times\, 10^4$ and $\times\, 10^3$, respectively, for scaling in (**B**) and (**C**).

REFERENCES AND NOTES

1. F. A. Lindemann, Über die Berechnung molekularer Eigenfrequenzen. *Phys. Z* **11**, 609–612 (1910).

2. M. Born, Thermodynamics of crystals and melting. *J. Chem. Phys.* **7**, 591–603 (1939).

3. Y. I. Frenkel, *Kinetic Theory of Liquids* (Dover, 1955).

4. D. Kuhlmann-Wilsdorf. Theory of melting. *Phys. Rev.* **140**, A1599–A1610 (1965).

5. J. Wang, J. Li, S. Yip, D. Wolf, S. Phillpot, Unifying two criteria of Born: Elastic instability and melting of homogeneous crystals. *Phys. A* **240**, 396–403 (1997).

6. J. G. Dash, History of the search for continuous melting. *Rev. Mod. Phys.* **71**, 1737–1743 (1999).

7. R. W. Cahn, Melting from within. *Nature* **413**, 582–583 (2001).

8. B. Rethfeld, K. Sokolowski-Tinten, D. von der Linde, S. I. Anisimov, Ultrafast thermal melting of laser-excited solids by homogeneous nucleation. *Phys. Rev. B* **65**, 092103 (2002).

9. S.-N. Luo, T. J. Ahrens, T. Çağın, A. Strachan, W. A. Goddard III, D. C. Swift, Maximum superheating and undercooling: Systematics, molecular dynamics simulations, and dynamic experiments. *Phys. Rev. B* **68**, 134206 (2003).

10. D. S. Ivanov, L. V. Zhigilei, Kinetic limit of heterogeneous melting in metals. *Phys. Rev. Lett.* **98**, 195701 (2007).

11. S. Williamson, G. Mourou, J. C. M. Li, Time-resolved laser-induced phase transformation in aluminum. *Phys. Rev. Lett.* **52**, 2364–2367 (1984).

12. B. J. Siwick, J. R. Dwyer, R. E. Jordan, R. J. D. Miller, An atomic-level view of melting using femtosecond electron diffraction. *Science* **302**, 1382–1385 (2003).

13. D. S. Ivanov, L. V. Zhigilei, Effect of pressure relaxation on the mechanisms of short-pulse laser melting, *Phys. Rev. Lett.* **91**, 105701 (2003).

14. Z. Lin, L. V. Zhigilei, Time-resolved diffraction profiles and atomic dynamics in short-pulse laser-induced structural transformations: Molecular dynamics study. *Phys. Rev. B* **73**, 184113 (2006).

15. J. R. Dwyer, C. T. Hebeisen, R. Ernstorfer, M. Harb, V. B. Deyirmenjian, R. E. Jordan, R. J. D. Miller, Femtosecond electron diffraction: 'Making the molecular movie'. *Philos. Trans. R. Soc. A* **364**, 741–778 (2006).

16. J. R. Dwyer, R. E. Jordan, C. T. Hebeisen, M. Harb, R. Ernstorfer, T. Dartigalongue, R. J. D. Miller, Femtosecond electron diffraction: An atomic perspective of condensed phase dynamics. *J. Modern Opt.* **54**, 905–922 (2007).

17. R. Ernstorfer, M. Harb, C. T. Hebeisen, G. Sciaini, T. Dartigalongue, R. J. D. Miller, The formation of warm dense matter: Experimental evidence for electronic bond hardening in gold. *Science* **323**, 1033–1037 (2009).

18. P. Musumeci, J. T. Moody, C. M. Scoby, M. S. Gutierrez, M. Westfall, Laser-induced melting of a single crystal gold sample by time-resolved ultrafast relativistic electron diffraction. *Appl. Phys. Lett.* **97**, 063502 (2010).

19. Z. Lin, E. Leveugle, E. M. Bringa, L. V. Zhigilei, Molecular dynamics simulation of laser melting of nanocrystalline Au. *J. Phys. Chem. C* **114**, 5686–5699 (2010).

20. Y. Giret, N. Naruse, S. L. Daraszewicz, Y. Murooka, J. Yang, D. M. Duffy, A. L. Shluger, K. Tanimura, Determination of transient atomic structure of laser-excited materials from time-resolved diffraction data. *Appl. Phys. Lett.* **103**, 253107 (2013).

21. S. L. Daraszewicz, Y. Giret, N. Naruse, Y. Murooka, J. Yang, D. M. Duffy, A. L. Shluger, K. Tanimura, Structural dynamics of laser-irradiated gold nanofilms. *Phys. Rev. B* **88**, 184101 (2013).

22. M. Z. Mo, Z. Chen, R. K. Li, M. Dunning, B. B. L. Witte, J. K. Baldwin, L. B. Fletcher, J. B. Kim, A. Ng, R. Redmer, A. H. Reid, P. Shekhar, X. Z. Shen, M. Shen, K. Sokolowski-Tinten, Y.

Y. Tsui, Y. Q. Wang, Q. Zheng, X. J. Wang, S. H. Glenzer, Heterogeneous to homogeneous melting transition visualized with ultrafast electron diffraction. *Science* **360**, 1451–1455 (2018).

23. Z. Chen, M. Mo, L. Soulard, V. Recoules, P. Hering, Y. Y. Tsui, S. H. Glenzer, A. Ng, Interatomic potential in the nonequilibrium warm dense matter regime. *Phys. Rev. Lett.* **121**, 075002 (2018).

24. T. A. Assefa, Y. Cao, S. Banerjee, S. Kim, D. Kim, H. Lee, S. Kim, J. H. Lee, S.-Y. Park, I. Eom, J. Park, D. Nam, S. Kim, S. H. Chun, H. Hyun, K. S. Kim, P. Juhas, E. S. Bozin, M. Lu, C. Song, H. Kim, S. J. L. Billinge, I. K. Robinson, Ultrafast x-ray diffraction study of melt-front dynamics in polycrystalline thin films. *Sci. Adv.* **6**, eaax2445 (2020).

25. Z. Chen, Y. Y. Tsui, M. Z. Mo, R. Fedosejevs, T. Ozaki, V. Recoules, P. A. Sterne, A. Ng, Electron kinetics induced by ultrafast photoexcitation of warm dense matter in a 30-nm-thick foil. *Phys. Rev. Lett.* **127**, 097403 (2021).

26. M. Mo, Z. Chen, S. Glenzer, Ultrafast visualization of phase transitions in nonequilibrium warm dense matter. *MRS Bull.* **46**, 694–703 (2021).

27. J. Hohlfeld, S.-S. Wellershoff, J. Güdde, U. Conrad, V. Jähnke, E. Matthias, Electron and lattice dynamics following optical excitation of metals. *Chem. Phys.* **251**, 237–258 (2000).

28. Z. Lin, L. V. Zhigilei, Thermal excitation of d band electrons in Au: Implications for laser-induced phase transformations. *Proc. SPIE* **6261**, 62610U (2006).

29. Z. Lin, L. V. Zhigilei, V. Celli, Electron-phonon coupling and electron heat capacity of metals under conditions of strong electron-phonon nonequilibrium. *Phys. Rev. B* **77**, 075133 (2008).

30. V. Recoules, J. Clérouin, G. Zérah, P. M. Anglade, S. Mazevet, Effect of intense laser irradiation on the lattice stability of semiconductors and metals. *Phys. Rev. Lett.* **96**, 055503 (2006).

31. J. W. Arblaster, Thermodynamic properties of gold. *J. Phase Equilibria Diffus.* **37**, 229–245 (2016).

32. M. V. Shugaev, M. He, Y. Levy, A. Mazzi, A. Miotello, N. M. Bulgakova, L. V. Zhigilei, Laser-induced thermal processes: Heat transfer, generation of stresses, melting and solidification, vaporization and phase explosion, in *Handbook of Laser Micro- and Nano-Engineering*, K. Sugioka, Ed. (Springer, 2021), pp. 83–163.

33. S. Mazevet, J. Clérouin, V. Recoules, P. M. Anglade, G. Zerah, Ab-initio simulations of the optical properties of warm dense gold. *Phys. Rev. Lett.* **95**, 085002 (2005).

34. N. Medvedev, I. Milov, Electron-phonon coupling in metals at high electronic temperatures. *Phys. Rev. B* **102**, 064302 (2020).

35. Y. V. Petrov, N. A. Inogamov, K. P. Migdal, Thermal conductivity and the electron-ion heat transfer coefficient in condensed media with a strongly excited electron subsystem. *JETP Lett.* **97**, 20–27 (2013).

36. B. Holst, V. Recoules, S. Mazevet, M. Torrent, A. Ng, S. Kirkwood, V. Sametoglu, M. Reid, Y. Y. Tsui, *Ab initio* model of optical properties of two-temperature warm dense matter. *Phys. Rev. B* **90**, 035121 (2014).

37. A. M. Brown, R. Sundararaman, P. Narang, W. A. Goddard III, H. A. Atwater, *Ab initio* phonon coupling and optical response of hot electrons in plasmonic metals, *Phys. Rev. B* **94**, 075120 (2016).

38. L. Waldecker, R. Bertoni, R. Ernstorfer, J. Vorberger, Electron-phonon coupling and energy flow in a simple metal beyond the two-temperature approximation. *Phys. Rev. X* **6**, 021003 (2016).

39. N. A. Smirnov, Copper, gold, and platinum under femtosecond irradiation: Results of first-principles calculations. *Phys. Rev. B* **101**, 094103 (2020).

40. Y. Li, P. Ji, *Ab initio* calculation of electron temperature dependent electron heat capacity and electron-phonon coupling factor of noble metals. *Comput. Mater. Sci.* **202**, 110959 (2022).

41. Here, the term “lattice temperature” does not imply the preservation of the crystal structure of the film but simply follows the convention used in the description of the results of TTM calculations. In the TTM-MD model, the “lattice temperature” is calculated from the kinetic energy of atomic motion, regardless of the phase state of the material.

42. The magnitude of the laser-induced stresses is characterized in this paper by pressure, defined as negative one third of the first invariant of the stress tensor, $P = -\left(\sigma_{xx} + \sigma_{yy} + \sigma_{zz}\right)/3$. This does not imply the hydrostatic stress conditions, though, as the value of P may include different contributions from different diagonal components of the stress tensor under conditions when the film undergoes uniaxial expansion and compression.

43. Yu. B. Zel’dovich, Yu. P. Raizer, *Physics of Shock Waves and High-Temperature Hydrodynamic Phenomena* (Dover Publications, 2002).

44. S. D. Brorson, A. Kazeroonian, J. S. Moodera, D. W. Face, T. K. Cheng, E. P. Ippen, M. S. Dresselhaus, G. Dresselhaus, Femtosecond room-temperature measurement of the electron-phonon coupling constant λ in metallic superconductors, *Phys. Rev. Lett.* **64**, 2172–2175 (1990).

45. H. E. Elsayed-Ali, T. Juhasz, G. O. Smith, W. E. Bron, Femtosecond thermoreflectivity and thermotransmissivity of polycrystalline and single-crystalline gold films. *Phys. Rev. B* **43**, 4488–4491 (1991).

46. R. H. M. Groeneveld, R. Sprik, A. Lagendijk. Femtosecond spectroscopy of electron-electron and electron-phonon energy relaxation in Ag and Au. *Phys. Rev. B* **51**, 11433–11445 (1995).

47. J. L. Hostetler, A. N. Smith, D. M. Czajkowsky, P. M. Norris, Measurement of the electron-phonon coupling factor dependence on film thickness and grain size in Au, Cr, and Al. *Appl. Optics* **38**, 3614–3620 (1999).

48. W. Wang, D. G. Cahill, Limits to thermal transport in nanoscale metal bilayers due to weak electron-phonon coupling in Au and Cu. *Phys. Rev. Lett.* **109**, 175503 (2012).

49. A. Giri, J. T. Gaskins, B. M. Foley, R. Cheaito, P. E. Hopkins, Experimental evidence of excited electron number density and temperature effects on electron-phonon coupling in gold films. *J. Appl. Phys.* **117**, 044305 (2015).

50. L. Guo, X. Xu, Ultrafast spectroscopy of electron-phonon coupling in gold. *J. Heat Transfer* **136**, 122401 (2014).

51. A. Nakamura, T. Shimojima, M. Nakano, Y. Iwasa, K. Ishizaka, Electron and lattice dynamics of transition metal thin films observed by ultrafast electron diffraction and transient optical measurements. *Struct. Dyn.* **3**, 064501 (2016).

52. The effective diameter *D* of a crystalline region is defined as the diameter of a spherical crystalline particle with the same number of atoms *n* as in the crystalline region. It is calculated as $D = a\sqrt[3]{3n/(2\pi)}$, where *a* is the lattice parameter of Au fcc crystal at a current temperature of the liquid-crystal coexistence system.

53. V. V. Zhakhovskii, N. A. Inogamov, Y. V. Petrov, S. I. Ashitkov, K. Nishihara, Molecular dynamics simulation of femtosecond ablation and spallation with different interatomic potentials. *Appl. Surf. Sci.* **255**, 9592–9596 (2009).

54. A better agreement between the experimental melting times and the TTM calculations are achieved in (*22*) by (i) neglecting the possibility of the homogeneous melting and (ii) making a physically questionable assumption that the melting front can only move if the temperature at the front exceeds $T_\mathrm{m} + \Delta H_\mathrm{m}/c_\mathrm{p} = 1780\ \mathrm{K}\ = 1.33T_\mathrm{m}$, evaluated with $\Delta H_\mathrm{m} = 66\ \mathrm{kJ/kg}$, $c_\mathrm{p} = 2.9\ \mathrm{MJ\ m^{-3}\ K^{-1}}$, and $T_\mathrm{m} = 1340\ \mathrm{K}$ assumed in (*22*). With this assumption, the melting would not occur in a film thermally equilibrated at any temperature below $1.33\ T_\mathrm{m}$. That is, the melting front would not propagate, and the film would remain in the state of superheated crystal-liquid coexistence indefinitely at any temperature in the range from T_m to $1.33\ T_\mathrm{m}$.

55. D. M. Wood, N. W. Ashcroft, Quantum size effects in the optical properties of small metallic particles. *Phys. Rev. B* **25**, 6255–6274 (1982).

56. G. V. Hartland, Optical studies of dynamics in noble metal nanostructures. *Chem. Rev.* **111**, 3858–3887 (2011).

57. A. Arbouet, C. Voisin, D. Christofilos, P. Langot, N. D. Fatti, F. Vallée, J. Lermé, G. Celep, E. Cottancin, M. Gaudry, M. Pellarin, M. Broyer, M. Maillard, M. P. Pileni, and M. Treguer, Electron-phonon scattering in metal clusters, *Phys. Rev. Lett.* **90**, 177401 (2003).

58. D. Mongin, P. Maioli, J. Burgin, P. Langot, E. Cottancin, S. D'Addato, B. Canut, M. Treguer, A. Crut, F. Vallée, N. Del Fatti, Ultrafast electron-lattice thermalization in copper and other noble metal nanoparticles. *J. Phys. Condens. Matter* **31**, 084001 (2019).

59. T. Q. Qiu, C. L. Tien, Size effects on nonequilibrium laser heating of metal films. *J. Heat Transfer.* **115**, 842–847 (1993).

60. U. B. Singh, C. Pannu, D. C. Agarwal, S. Ojha, S. A. Khan, S. Ghosh, D. K. Avasthi, Large electronic sputtering yield of nanodimensional Au thin films: Dominant role of thermal conductivity and electron phonon coupling factor. *J. Appl. Phys.* **121**, 095308 (2017).

61. W. G. Ma, H. D. Wang, X. Zhang, W. Wang, Experiment study of the size effects on electron-phonon relaxation and electrical resistivity of polycrystalline thin gold films. *J. Appl. Phys.* **108**, 064308 (2010).

62. T. Vasileiadis, L. Waldecker, D. Foster, A. Da Silva, D. Zahn, R. Bertoni, R. E. Palmer, R. Ernstorfer, Ultrafast heat flow in heterostructures of Au nanoclusters on thin films: Atomic disorder induced by hot electrons. *ACS Nano* **12**, 7710–7720 (2018).

63. D. M. Riffe, X. Y. Wang, M. C. Downer, D. L. Fisher, T. Tajima, J. L. Erskine, R. M. More, Femtosecond thermionic emission from metals in the space-charge-limited regime. *J. Opt. Soc. Am. B* **10**, 1424–1435 (1993).

64. W. Wendelen, D. Autrique, A. Bogaerts, Space charge limited electron emission from a Cu surface under ultrashort pulsed laser irradiation. *Appl. Phys. Lett.* **96**, 051121 (2010).

65. M. V. Shugaev, C.-Y. Shih, E. T. Karim, C. Wu, L. V. Zhigilei, Generation of nanocrystalline surface layer in short pulse laser processing of metal targets under conditions of spatial confinement by solid or liquid overlayer. *Appl. Surf. Sci.* **417**, 54–63 (2017).

66. S. A. Lizunov, A. V. Bulgakov, E. E. B. Campbell, N. M. Bulgakova, Melting of gold by ultrashort laser pulses: Advanced two-temperature modeling and comparison with surface damage experiments. *Appl. Phys. A* **128**, 602 (2022).

67. D. S. Ivanov, L. V. Zhigilei, Combined atomistic-continuum modeling of short-pulse laser melting and disintegration of metal films. *Phys. Rev. B* **68**, 064114 (2003).

68. X. Y. Wang, D. M. Riffe, Y.-S. Lee, M. C. Downer, Time-resolved electron-temperature measurement in a highly excited gold target using femtosecond thermionic emission. *Phys. Rev. B* **50**, 8016–8019 (1994).

69. L. A. Zepeda-Ruiz, B. Sadigh, A. A. Chernov, T. Haxhimali, A. Samanta, T. Oppelstrup, S. Hamel, L. X. Benedict, J. L. Belof, Extraction of effective solid-liquid interfacial free energies for full 3D solid crystallites from equilibrium MD simulations. *J. Chem. Phys.* **147,** 194704 (2017).

70. D. Turnbull, Formation of crystal nuclei in liquid metals. *J. Appl. Phys.* **21**, 1022–1028 (1950).

71. C. Suárez, W. E. Bron, T. Juhasz, Dynamics and transport of electronic carriers in thin gold films. *Phys. Rev. Lett.* **75**, 4536–4539 (1995).

72. D. A. Thomas, Z. Lin, L. V. Zhigilei, E. L. Gurevich, S. Kittel, R. Hergenröder, Atomistic modeling of femtosecond laser-induced melting and atomic mixing in Au film – Cu substrate system. *Appl. Surf. Sci.* **255**, 9605–9612 (2009).

73. J. R. Morris, X. Song, The melting lines of model systems calculated from coexistence simulations. *J. Chem. Phys.* **116**, 9352–9358 (2002).

74. C. Alves, J. S. Pedersen, C. L. P. Oliveira, Calculation of two-dimensional scattering patterns for oriented systems. *J. Appl. Cryst.* **50**, 840–850 (2017).

75. T. Egami, S. J. L. Billinge, *Underneath the Bragg Peaks: Structural Analysis of Complex Materials* (Elsevier, 2003).

76. C. Colliex, J. M. Cowley, S. L. Dudarev, M. Fink, J. Gjønnes, R. Hilderbrandt, A. Howie, D. F. Lynch, L. M. Peng, G. Ren, A. W. Ross, V. H. Smith Jr, J. C. H. Spence, J. W. Steeds, J. Wang, M. J. Whelan, B. B. Zvyagin, Electron Diffraction, in *International Tables for Crystallography* (International Union of Crystallography, 2006), vol. C, chap. 4.3, pp. 259–429.

77. https://compmat.org/2D-diffraction/

78. D. Turnbull, J. C. Fisher, Rate of nucleation in condensed systems. *J. Chem. Phys.* **17**, 71–73 (1949).

79. J. M. Howe, *Interfaces in Materials: Atomic Structure, Thermodynamics and Kinetics of Solid-Vapor, Solid-Liquid and Solid-Solid Interfaces* (John Wiley & Sons Inc., 1997).

80. A. Stukowski, Visualization and analysis of atomistic simulation data with OVITO–the Open Visualization Tool. *Model. Simul. Mater. Sci. Eng.* **18**, 015012 (2009).

81. X. Sedao, M. V. Shugaev, C. Wu, T. Douillard, C. Esnouf, C. Maurice, S. Reynaud, F. Pigeon, F. Garrelie, L. V. Zhigilei, J.-P. Colombier, Growth twinning and generation of high-frequency surface nanostructures in ultrafast laser-induced transient melting and resolidification. *ACS Nano* **10**, 6995–7007 (2016).

82. F. Celestini, J.-M. Debierre, Measuring kinetic coefficients by molecular dynamics simulation of zone melting. *Phys. Rev. E* **65**, 041605 (2002).

83. N. M. Bulgakova, R. Stoian, A. Rosenfeld, I. V. Hertel, W. Marine, E. E. B Campbell. A general continuum approach to describe fast electronic transport in pulsed laser irradiated materials: The problem of Coulomb explosion. *Appl. Phys. A* **81**, 345–356 (2005).

84. E. M. Logothetis, P. L. Hartman, Laser-induced electron emission from solids: Many-photon photoelectric effects and thermionic emission. *Phys. Rev.* **187**, 460–474 (1969).

85. J. P. Girardeau-Montaut, C. Girardeau-Montaut, Theory of ultrashort nonlinear multiphoton photoelectric emission from metals. *Phys. Rev. B* **51**,13560–13567 (1995).

86. L. A. DuBridge, A further experimental test of Fowler's theory of photoelectric emission. *Phys. Rev.* **39**, 108–118 (1932).

87. P. B. Johnson,R. W. Christy, Optical constants of the noble metals. *Phys. Rev. B* **6**, 4370–4379 (1972).

88. D. S. Ivanov, “Computational investigation of short pulse laser melting of metal targets,” thesis, University of Virginia, Charlottesville, VA (2005).
